# Identification of approved and investigational drugs that inhibit hypoxia-inducible factor-1 signaling

**DOI:** 10.18632/oncotarget.6995

**Published:** 2016-01-23

**Authors:** Chia-Wen Hsu, Ruili Huang, Thai Khuc, David Shou, Joshua Bullock, Suzanne Grooby, Sue Griffin, Chaozhong Zou, Annette Little, Holly Astley, Menghang Xia

**Affiliations:** ^1^ Division of Pre-Clinical Innovation, National Center for Advancing Translational Sciences, National Institutes of Health, Bethesda, MD, USA; ^2^ Horizon Discovery Ltd., Waterbeach, Cambridge, UK; ^3^ American Type Culture Collection, Gaithersburg, MD, USA

**Keywords:** hypoxia inducible factor, cancer, genome editing, drug, high-throughput screening

## Abstract

One of the requirements for tumor development is blood supply, most often driven by hypoxia-induced angiogenesis. Hypoxia induces the stabilization of hypoxia-inducible factor-1 alpha (HIF-1α), which induces expression of an angiogenic factor, vascular endothelial growth factor (VEGF). The purpose of this study is to validate a new screening platform combined with orthogonal assays to rapidly identify HIF-1 inhibitors and to evaluate the effectiveness of approved drugs on modulating HIF-1 signaling.

We generated an endogenous HIF-1α–NanoLuc luciferase reporter allele in the human HCT116 colon cancer cell line using genome editing and screened a panel of small interfering RNAs (siRNAs) to 960 druggable targets and approximately 2,500 drugs on a quantitative high-throughput screening (qHTS) platform. Selected compounds were further investigated with secondary assays to confirm their anti-HIF activity and to study their mode of action. The qHTS assay identified over 300 drugs that inhibited HIF-1α-NanoLuc expression. The siRNA screening results supported the effectiveness of several target-specific inhibitors. Moreover, the identified HIF-1 inhibitors, such as mycophenolate mofetil, niclosamide, and trametinib, were able to suppress cancer cell proliferation and angiogenesis. Our study indicates that blocking the mitogen-activated protein kinase (MAPK) and phosphoinositol 3-kinase (PI3K) pathways effectively inhibits hypoxia-induced HIF-1α accumulation and HIF-1α transactivation and that proteasome inhibitors induce accumulation and decrease transcriptional activity of HIF-1α. These findings underline the importance of developing a battery of robust assay platforms and confirmation studies that focus on endogenous protein targets so that only relevant and reliable data will be taken into pre-clinical and clinical studies.

## INTRODUCTION

Hypoxia-inducible factors (HIFs) have a crucial role in cancer development, progression, and metastasis [1]. As a tumor develops, the escalating number of cells increases the rate of oxygen consumption at the tumor site and creates hypoxic stress. Hypoxia results in the stabilization of the labile HIF-1 subunit HIF-1α and the induction of HIF-1 target gene transcription. The increased expression of HIF-1 target genes, including vascular endothelial growth factor (*VEGF*) and other angiogenic genes, results in blood vessel formation and tumor expansion. Thus, HIF-1α is a potential target for inhibition of both tumor-mediated angiogenesis and other aspects of tumor development, such as metabolic alterations that further increase the proliferation of tumor cells. Based on mechanism of action, HIF- 1α inhibitors can be categorized into inhibitors of HIF- 1– DNA- binding activity, inhibitors of *HIF1A* messenger RNA (mRNA) expression, inhibitors of HIF-1α protein translation, inhibitors of HIF-1α transcriptional activity, and activators of the prolyl-hydroxylase-driven HIF-1α degradation pathway [2]. Over 20 HIF- 1 inhibitors, including topotecan (Hycamtin), vorinostat (Zolinza) and YC-1, which are approved anti-cancer drugs, have been tested in clinical trials, or are being investigated in pre-clinical studies [3].

The translation of pre-clinical research findings to clinical research, particularly to oncology drug development, is challenging owing to the highly dynamic and heterogeneous nature of cancer cells [4]. The generation of reliable data requires physiologically relevant *in vitro* and *in vivo* models, robust assay technologies, and well-executed confirmation and validation studies. Clinically approved drugs and investigational drugs are increasingly being assessed for their anticancer properties primarily because these compounds have already been tested for toxicity, pharmacokinetics, and drug-drug interactions. Thus, there is a need to develop a robust, reliable, physiologically relevant, and high-throughput screening (HTS)-compatible platform that can assess the effects of drugs on endogenous targets. HTS is a popular route for drug discovery, drug development, and target identification. Quantitative HTS (qHTS), a titration-based approach that tests multiple compound concentrations, is capable of generating concentration-response curves for thousands of compounds measured in a single experiment [5], greatly reducing false positive and false negative rates [6]. Robust statistical methods and secondary assay strategies can be employed to further improve data reliability.

Current technologies for high-throughput and high-content screening often involve the use of target proteins that are not expressed from their endogenous promoters and the use of surrogate markers of activity, both approaches can yield non-physiological results. However, using a recombinant adeno-associated virus (rAAV) genome editing platform, one can precisely knock a reporter gene into an allele of interest, permitting evaluation of genes and proteins at physiologically relevant levels. Nano Luciferase (NanoLuc) is a small (< 20 kDa), bright (> 150-fold of firefly luciferase) reporter with glow-type luminescence (approximate half-life: 120 minutes) [7] that can be used to accurately measure low levels of protein expression from endogenous promoters. In this study, we have used rAAV genome editing technology to generate a HCT116 human colon cancer HIF-1α–NanoLuc reporter cell line. This reporter cell line was used in a qHTS platform to evaluate the effect of 2,457 clinically-used and investigational drugs in the NCATS Pharmaceutical Collection (NPC) [8] on hypoxia-induced HIF-1α–NanoLuc protein accumulation.

## RESULTS

### Identification of HIF-1 inhibitors using a qHTS platform

**Figure 1:**
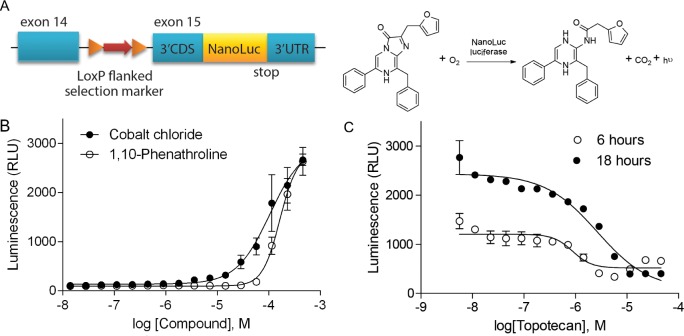
Validation of HIF-1α–NanoLuc assay in 1536-well qHTS formats (**A**) Schematic of the endogenous targeted *HIF1A* allele of the HIF-1α–NanoLuc luciferase protein reporter cell line and the NanoLuc luciferase reaction. Exon numbers refer to transcript ENST00000337138. (**B**) Concentration-response curves of cobalt chloride and 1, 10-phenathroline under normoxic condition. (**C**) Concentration-response curves of topotecan under hypoxic condition (1% O_2_) for six and eighteen hours. Data are expressed as mean ± standard deviation (SD) from four experiments.

The HIF-1α–NanoLuc reporter cell line was generated using rAAV-mediated genome editing technology to introduce a NanoLuc reporter sequence downstream of and in frame with the last coding exon of the *HIF1A* gene (Figure 1A). The function of this reporter cell line was validated in 96-well and 1536-well formats using known factors such as low oxygen concentration and HIF-1 modulators that alter hypoxia signaling (Figure 1 and Figure S1). Under hypoxic conditions the relative luminescence unit (RLU) values measured from the HIF- 1α-NanoLuc reporter were proportional to the HIF- 1α-NanoLuc protein levels measured by western blotting (Figure S1B). These initial experiments indicated that treatment for 18 hours with topotecan under hypoxic condition robustly and consistently reduced hypoxia-induced HIF-1α–NanoLuc expression with a Z’ factor value of 0.58, while a 6-hour incubation yielded a lower Z’ factor of 0.38. Thus, topotecan as the positive control and an 18-hour incubation time in a hypoxic (1% O_2_) chamber were selected for the qHTS of HIF-1 inhibitors.

The HIF-1α–NanoLuc assay was used to identify potential HIF-1α inhibitors from the NCATS Pharmaceutical Collection (NPC), which contains clinically-approved and investigational drugs. The average signal-to-background (S/B) ratio, coefficient of variation (CV) value, and Z’ factor from the primary screen of 20 assay plates were 3.4, 6%, and 0.66 respectively. Three hundred and five compounds decreased hypoxia-induced HIF-1α–NanoLuc expression in HCT116 cells after 18 hours of compound treatment. Twenty-two compounds previously reported as HIF-1 inhibitors were identified from our HIF-1α–NanoLuc screen (Table S1). Anthracycline chemotherapeutic agents, anti-metabolic nucleobase analogs, dihydropyridine calcium channel blockers, cardiac glycosides, and quinolone chemotherapeutic were identified as HIF-1α–NanoLuc inhibitors. Moreover the HIF-1α–NanoLuc assay identified several pharmacological inhibitors that target the RAS–RAF–MEK–ERK and PI3K–AKT–mTOR signaling pathways (Table S2).

### Identification of drug targets using an siRNA screen

**Figure 2:**
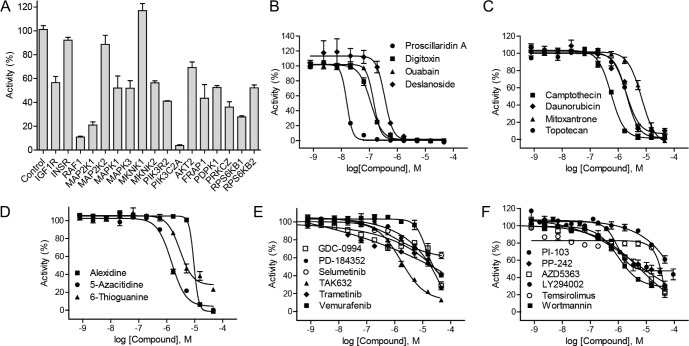
Identification of HIF-1 pathway inhibitors by HIF-1α–NanoLuc assay (**A**) Luciferase signals of HIF-1α–NanoLuc reporter cell line reverse transfected with selected siRNA duplexes under hypoxic (1% O_2_) conditions. siRNA screening data were expressed as a percentage of the non-targeting control siRNA data, mean ± SD from three measurement. (**B**) Graph showing a concentration-dependent response of hypoxia-induced HIF-1α-NanoLuc activity to cardiac glycosides: deslanoside, digitoxin, ouabain, and proscillaridin A. (**C**) Graph showing a concentration-dependent response of hypoxia-induced HIF-1α-NanoLuc activity to of topoisomerase inhibitors: daunorubicin, campthecin, mitoxantrone, and topotecan. (**D**) Graph showing a concentration-dependent response of hypoxia-induced HIF-1α-NanoLuc activity to nucleoside analogs: alexidine, 5-azacitidine, and thioguanine. (**E**) Graph showing a concentration-dependent response of hypoxia-induced HIF-1α-NanoLuc activity to RAS–RAF–MEK–ERK pathway inhibitors: GDC-0994, PD-184352, selumetinib, TAK632, trametinib, and vemurafenib. (**F**) Graph showing a concentration-dependent response of hypoxia-induced HIF-1α-NanoLuc activity to PI3K–AKT–mTOR pathway inhibitors: LY294002, PI-103, PP-242, temsirolimus, and wortmannin. Confirmatory screening data of small molecule inhibitors were expressed as mean ± SD from three measurements.

To identify drug targets that are related to HIF-1 signaling pathway, the HIF-1α–NanoLuc reporter cell line was used to screen a 960 siRNA druggable target library under hypoxic conditions. Down-regulation of mRNAs in the RAS–RAF–MEK–ERK pathways, including *RAF1*, *MAP2K1*, *MAP2K2*, *MAPK3* and *MKNK2*, resulted in decreased hypoxia-induced HIF-1α–NanoLuc reporter gene expression (Figure 2A and Table S2). siRNAs targeting *RAF1* and *MAP2K1* were the most effective with more than 80% efficacy, whereas inhibition by siRNAs targeting *MAP2K2* and *MKNK1* had no effect on HIF-1α–NanoLuc activity. siRNAs that targeted components of the PI3K–AKT–mTOR and PKC pathways also inhibited HIF-1α–NanoLuc activity under hypoxic conditions (Figure 2A and Table S2). A substantial inhibitory effect was mediated by a siRNAs targeting *PIK3C2A*. Silencing of insulin-like growth factor 1 receptor (*IGF1R*) also resulted in decreased in HIF-1α–NanoLuc activity (Figure 2A and Table S2).

The siRNA screen data also indicated that a number of canonical pathways, including the pyrimidine ribonucleotide salvage pathways, the pyridoxal 5′-phosphate salvage pathway, and the nuclear factor-kappa-B (NF-kB) signaling pathway, are potential modulators of HIF-1α–NanoLuc activity (Table S3). The top disease and biological function networks identified from the siRNA screen that affected HIF-1α–NanoLuc activity include post-transcriptional modification, post-translational modification, protein degradation, and cancer associated networks (Table S4). Consistent with the findings from an independent study that reported an siRNA screen in melanoma cells [9], our results revealed that silencing of the three DNA damage response (DDR) factors—*ATM*, checkpoint kinase 1 (*CHEK1*) and checkpoint kinase 2 (*CHEK2*)— led to altered HIF-1α-NanoLuc expression levels under hypoxic conditions and the three genes are involved in several signaling pathways and networks identified by the siRNA screen (Table S3 and S4). The siRNA screen also identified siRNAs that resulted in increased activity of HIF-1α–NanoLuc under hypoxic conditions, such as siRNAs targeting sirtuins (*SIRT*s) and fms-related typrosine kinase 1 (*FLT1*) (Table S5). The HIF-1α–NanoLuc activity was significantly down-regulated by siRNAs targeting fibroblast growth factor receptor 2 (*FGFR2*) and phosphoglycerate kinase 1 (*PGK1*) (Table S6).

### Confirmation of identified HIF-1 inhibitors

From the primary screen, 40 HIF-1α–NanoLuc inhibitors selected based on potency (≤ 10 uM), efficacy (≥ 50%), goodness of curve fit (curve class of −1.1, −1.2, −2.1, and −2.2) (Figure S2), or biological interest, were re-tested in the HIF-1α–NanoLuc HCT116 cells and counter screened in the hypoxia response element-beta-lactamase (HRE–bla) reporter gene assay [6] to eliminate potential assay artifacts caused by non-specific interactions or compound cytotoxicity (Table 1 and Table S7). All of the 40 HIF-1α–NanoLuc inhibitors reduced HIF-1α–NanoLuc levels in a concentration-dependent manner and 37 of these compounds (93%) also decreased HRE–bla transactivation activity (Table S7). Cardiac glycosides and other inhibitors of transcription or translation such as actinomycin and cycloheximide suppressed hypoxia-induced HIF-1α–NanoLuc expression with sub-micromolar potency (Figure 2B and Table S7). The proteasome inhibitors bortezomib and carfilzomib, which potently induced accumulation of HIF-1α–NanoLuc proteins in the HCT116 cells, inhibited the transcriptional activity of HRE–bla in the ME-180 cells (Figure S3).

**Table 1:** Summary of potency and efficacy values of the selected HIF-1 inhibitors

Compound Name	Chemical Structure	HIF-1α-NanoLuc IC_50_, μM (Efficacy, %)	HRE-bla IC_50_, μM (Efficacy, %)	72-hr Viability HCT116 IC_50_, μM (Efficacy, %)	72-hr Viability ME-180 IC_50_, μM (Efficacy, %)	Angiogenesis IC_50_, μM (Efficacy, %)
5-Azacitidine	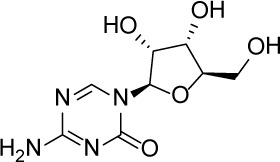	1.49 ± 0.17 (98 ± 4)	11.01 ± 0.75 (94 ± 2)	13.27 ± 1.52 (90 ± 9)	18.65 ± 0.00 (51 ± 12)	4.06 ± 1.49 (80 ± 18)
Camptothecin	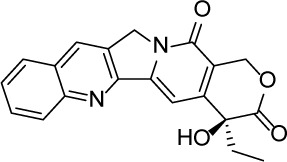	0.57 ± 0.04 (102 ± 5)	0.20 ± 0.06 (84 ± 1)	1.00 ± 0.29 (59 ± 9)	0.04 ± 0.00 (100 ± 2)	0.00027 ± 0.00031 (134 ± 36)
Mitoxantrone	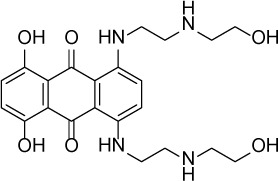	7.65 ± 2.13 (110 ± 5)	0.86 ± 0.52 (99 ± 10)	11.10 ± 4.79 (80 ± 5)	0.11 ± 0.03 (97 ± 4)	0.004 ± 0.003 (101 ± 9)
Mycophenolate mofetil	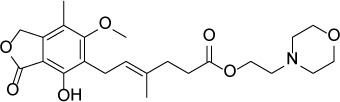	1.58 ± 0.45 (59 ± 5)	0.21 ± 0.05 (58 ± 4)	Inactive	1.82 ± 0.74 (81 ± 3)	0.11 ± 0.06 (93 ± 6)
Niclosamide	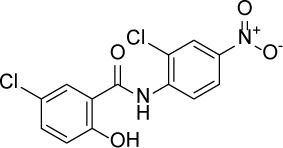	1.59 ± 0.40 (96 ± 5)	0.23 ± 0.05 (91 ± 6)	1.67 ± 0.46 (84 ± 6)	2.04 ± 0.00 (86 ± 1)	0.04 ± 0.03 (87 ± 14)
PI-103	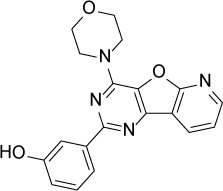	0.92 ± 0.06 (57 ± 10)	0.15 ± 0.02 (62 ± 3)	Inactive	0.37 ± 0.02 (85 ± 5)	0.32 ± 0.09 (80 ± 18)
PP-242	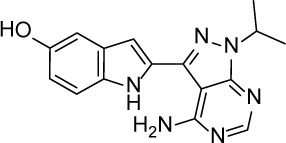	2.05 ± 1.12 (90 ± 11)	0.05 ± 0.02 (56 ± 6)	33.60 ± 6.28 (49 ± 20)	0.95 ± 0.22 (105 ± 7)	0.23 ± 0.11 (80 ± 18)
Selumetinib	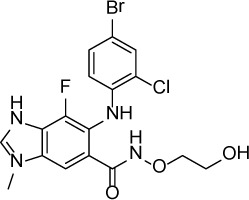	1.49 ± 1.30 (48 ± 10)	Inactive	22.83 ± 3.89 (105 ± 4)	0.27 ± 0.06 (36 ± 3)	0.01 ± 0.00 (92 ± 8)
Sunitinib	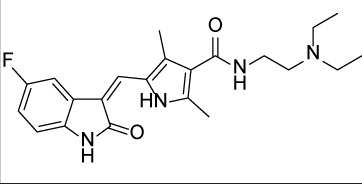	11.34 ± 3.71 (80 ± 15)	16.99 ± 3.17 (81 ± 32)	37.22 ± 0.00 (34 ± 31)	18.99 ± 4.34 (90 ± 7)	0.02 ± 0.01 (124 ± 1)
Topotecan	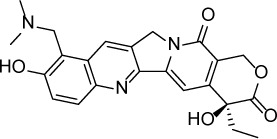	2.10 ± 0.24 (99 ± 6)	0.62 ± 0.38 (131 ± 9)	4.30 ± 1.20 (54 ± 22)	0.10 ± 0.01 (100 ± 2)	0.01 ± 0.00 (80 ± 18)
Trametinib	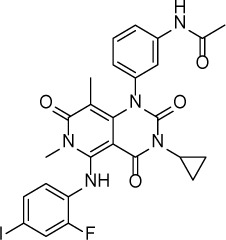	2.98 ± 4.64 (69 ± 5)	20.51 ± 8.11 (53 ± 9)	0.64 ± 0.11 (87 ± 3)	0.06 ± 0.02 (52 ± 7)	0.00005 ± 0.00005 (89 ± 19)

Potency and efficacy are defined as half maximal inhibitory concentration (IC_50_) and percentage of inhibition compared to DMSO controls, respectively.

A subset of 36 small molecule inhibitors that target the same regulators identified from the drug and siRNA screens were tested in the HIF-1α–NanoLuc assay to validate their effect on pathway inhibition. The topoisomerase inhibitor —camptothecin— was at least three-fold more potent than other topoisomerase inhibitors tested in our assays (Figure 2C). Nucleoside analogs such as 5-azacitidine, alexidine, and 6-thioguanine were confirmed to decrease HIF-1α–NanoLuc levels and HRE–bla transactivation activity (Figure 2D and Table S7). In addition to RAF inhibitors and MEK inhibitors identified from the primary screen, two more RAF inhibitors (TAK632 and vemurafenib), a new MEK inhibitor (trametinib), and an ERK inhibitor (GDC- 0994), were identified as HIF-1α inhibitors (Figure 2E and Table S2). To further support the anti-HIF activity of PI3K pathway inhibitors, three more PI3K inhibitors—PI-103, wortmannin, and LY294002, an additional AKT inhibitor AZD5363, and two more mTOR inhibitors—PI-103 and temsirolimus— were tested in the confirmatory screen (Figure 2F and Table S2). OSI-906, an inhibitor of IGF-1R, also suppressed HIF-1α–NanoLuc activity with an IC_50_ value of 6.23 ± 1.64 μM.

### Anti-proliferative effects of the HIF-1 inhibitors

**Figure 3:**
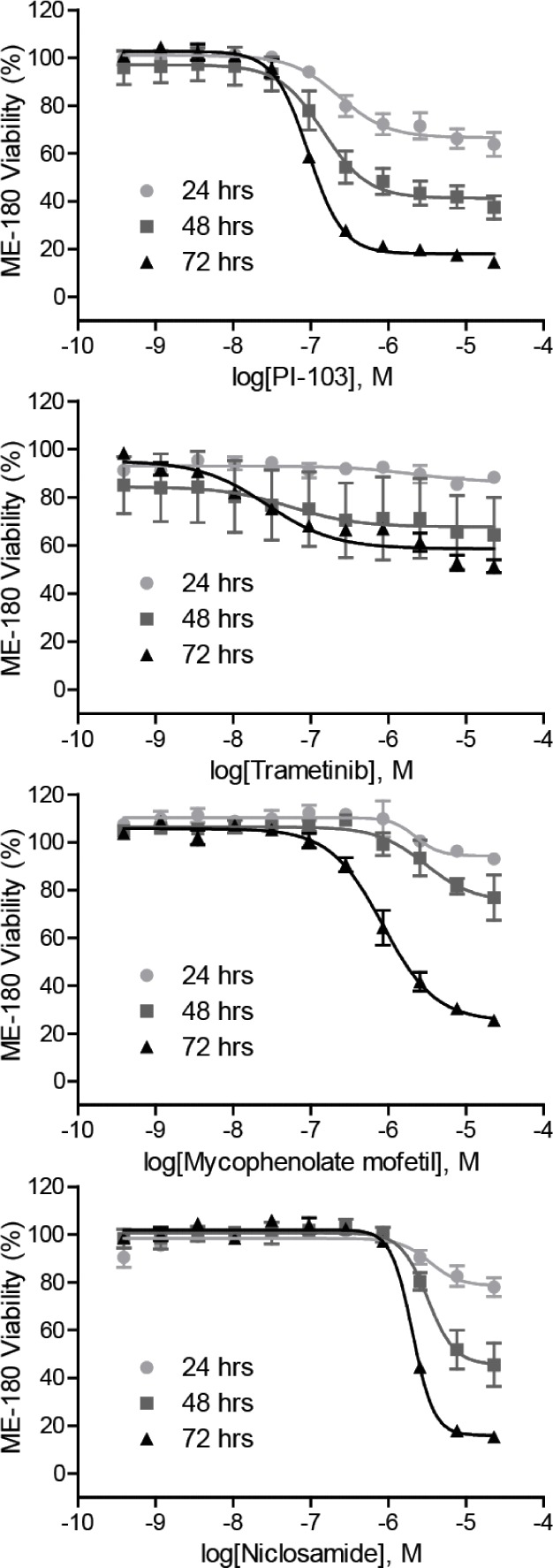
Anti-proliferative properties of the identified HIF-1 inhibitors Cell viability of ME-180 cells treated with increasing concentrations of PI-103, trametinib, mycophenolate mofetil, and niclosamide for 24, 48, and 72 hours under normoxic condition. Assay data are expressed as mean ± SD from three measurements.

Twenty confirmed HIF-1 inhibitors including cardiac glycosides; topoisomerase inhibitors; RAS–RAF–MEK–ERK pathway inhibitors; and AKT–PI3K–mTOR pathway inhibitors, were tested for their anti-proliferative activity over three days in human colon cancer HCT116 cells and human cervical cancer ME-180 cells (Table S8). A time-dependent decrease in cell viability was observed when ME-180 cells were treated with PI-103, trametinib, mycophenolate mofetil, and niclosamide (Figure 3). All compounds suppressed proliferation of ME-180 cells and 15 of these compounds reduced the viability of HCT116 by > 50% after three days of treatment (Table 1 and Table S8). Proscillaridin A, ouabain, and trametinib were the most potent compounds identified from the HCT116 viability screen, whereas all cardiac glycosides, camptothecin, daunorubicin, mitoxantrone, PI-103, PP- 242, and selumetinib robustly inhibited proliferation in ME-180 cells at submicromolar IC_50_ concentrations (Table 1 and Table S8).

### Effect of HIF-1α-NanoLuc inhibitors on angiogenesis inhibition

**Figure 4:**
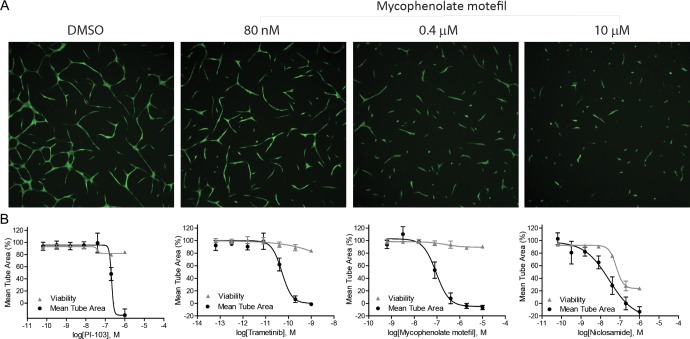
Anti-angiogenic properties of the identified HIF-1 inhibitors (**A**) Fluorescence images of GFP-expressing aortic endothelial cells in the presence of various concentrations of mycophenolate mofetil. (**B**) Tube formation and viability of co-cultures of human aortic endothelial cells and human mesenchymal stem cells after a 3-day exposure of PI-103, trametinib, mycophenolate mofetil, and niclosamide under normoxic condition. Assay data are expressed as mean ± SD from three measurements.

Ten representative HIF-1 inhibitors along with an angiogenesis inhibitor control, sunitinib, were tested for their ability to inhibit angiogenesis in an *in vitro* co-culture assay system (Table 1 and Figure 4). After a three-day exposure of test compounds, all compounds were able to completely block angiogenesis in the co-culture assay. Trametinib was the most potent angiogenesis inhibitor with a picomolar IC_50_ value. Mycophenolate mofetil and PI-103 caused half of maximum reduction in mean tube area at submicromolar concentrations. Despite cytotoxicity at high concentrations, niclosamide inhibited tube formation with an IC_50_ value of 0.04 ± 0.03 μM. It is very interesting to find that the identified HIF- 1 inhibitors were 2.7-fold to 59,600-fold more potent in preventing arotic endothelial cells from forming tubular structures than inhibiting HIF-1α–NanoLuc accumulation in HCT116 cells.

### Effect of HIF-1α inhibitors on the expression of hypoxia-targeted genes

**Figure 5:**
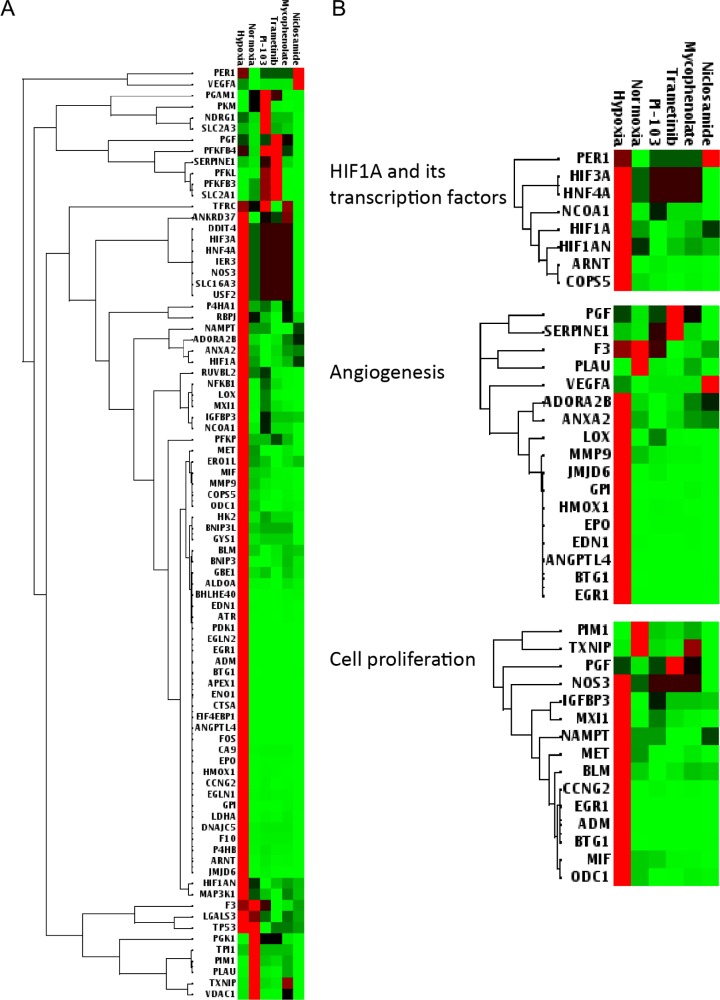
Heat maps of differentially expressed hypoxia signaling genes in ME-180 cells treated with the identified HIF-1 inhibitors (**A**) Clustergram of 84 genes related to human hypoxia signaling. (**B**) Clustergram of subsets of genes including *HIF1A* and its transcription factors (upper), angiogenesis (middle), and cell proliferation (lower). Cells were treated with controls (DMSO in 1% or 20% oxygen) or the identified HIF-1 inhibitors (mycophenolate mofetil, niclosamide, PI-103, and trametinib) at the corresponding IC_50_ concentrations established in the HIF-1α-NanoLuc assay under hypoxic (1% O_2_) condition. Red and green colors indicate higher and lower expression levels, respectively. Gene expression data are expressed as mean ΔΔCt values from three measurements.

In order to further study how the identified HIF-1α–NanoLuc inhibitors affect hypoxia signaling, ME-180 cells were exposed to hypoxia and treated with mycophenolate mofetil, niclosamide, PI-103, or trametinib at IC_50_ concentrations established in the HIF-1α–NanoLuc assay and gene expression changes were analyzed using a panel of 84 genes involved in human hypoxia signaling (Figure 5). The gene panel consists of *HIF1A*, co-transcription factors of HIF-1, other HIF-1 interacting proteins, and hypoxia responsive genes that regulate angiogenesis, apoptosis, cell proliferation, coagulation, DNA damage and repair, metabolism, and transcription. Seventy-one out of 84 genes, including annexin A2 (*ANXA2*) and vascular endothelial growth factor A (*VEGFA*), were significantly upregulated in ME-180 cells by hypoxia. As shown in Figure 5A, the four HIF-1α–NanoLuc inhibitors were able to suppress the expression of the majority of the hypoxia-induced genes in the gene panel. A closer look at the sub gene sets revealed that the four compounds induced similar gene expression changes in angiogenesis and cell proliferation (Figure 5B).

## DISCUSSION

Using an endogenous HIF-1α protein reporter assay, we have screened approximately 2,500 drugs and 960 siRNAs to druggable targets for their ability to modulate endogenous HIF-1α protein levels. The HCT116 cell line expressing an endogenous HIF-1α–NanoLuc fusion protein showed a robust response to factors known to modulate HIF-1 activity, including hypoxia, normoxia, hypoxia mimetic compounds (such as cobalt chloride and 1, 10-phenathroline) [10], HIF-1 inhibitors (such as cardiac glycosides, topoisomerase inhibitors, PI3K pathway inhibitors, and MAPK pathway inhibitors) [6, 11–13], siRNAs of HIF-1 regulators (such as targets in PI3K and MAPK pathways) [14], inhibitors of transcription (such as actinomycin) [15], inhibitors of translation (such as cycloheximide) [16], and proteasome inhibitors (such as bortezomib and carfilzomib) [17]. The paradoxical action of the two proteasome inhibitors to both increase HIF-1α–NanoLuc expression and inhibit HRE–bla activity (Figure S3) is consistent with a previous report that bortezomib could induce accumulation of HIF-1α proteins by proteasome inhibition, but inhibit HIF-1 transactivation activity by interacting with the C-terminal transactivation domain of HIF-1α [18].

Topoisomerase inhibitors are known to block HIF-1 signaling through multiple modes of action and exerted anti-proliferative effects on HCT116 and ME- 180 cells in our study and other studies. For example, topotecan has been shown to affect HIF-1α dynamics and transcriptional activity by blocking HIF-1α translation rather than by inducing DNA replication-introduced DNA damage or by inhibiting proteasome-mediated protein degradation [19]. Mitoxantrone can reduce HIF-1α levels in topoisomerase II-deficient HCT116 cells, probably through non-selective inhibition of protein translation [20]. In our study, topoisomerase inhibitors (camptothecin, daunorubicin, mitoxantrone, and topotecan) were more potent at blocking HIF-1α transactivation in ME-180 cells (IC_50_: 0.20–1.00 μM) than reducing the levels of the HIF-1α–NanoLuc protein in HCT116 cells (IC_50_ = 0.57–7.65 μM) (Table 1). In agreement with these findings, the topoisomerase inhibitors were more effective at inhibiting proliferation of the ME-180 cells (IC_50_: 0.04–0.17 μM) than the HCT116 cells (IC_50_ = 1.00–11.10 μM), a finding supported by a moderate correlation (R^2^ > 0.5) between anti-HIF activity and cancer cell viability. Moreover, camptothecin, mitoxantrone, and topotecan were 2100−, 1900−, and 210-fold more potent in inhibiting angiogenesis than HIF-1α–NanoLuc activity (Table 1). These results imply that HIF-1 inhibition might be one of the mechanisms through which topoisomerase inhibitors suppress cancer growth and angiogenesis. Future work should investigate the effectiveness of topoisomerase-dependent and -independent regulation of HIF-1 signaling on cancer proliferation and angiogenesis.

The anti-proliferative activity of MAPK pathway inhibitors was highly associated with compound potency in suppressing hypoxia-induced HIF-1α–NanoLuc protein accumulation. The RAS–RAF–MEK–ERK signaling cascade is a master regulator of HIF-1 signaling [21] in which HIF-1 transcription activity is promoted by ERK-mediated phosphorylation of HIF-1α protein [22]. In addition, PD98059, a MEK inhibitor, was previously shown to block HIF-1α protein synthesis and inhibit HIF-stimulated VEGF expression in human colon cancer cells treated with IGF-1 [23]. In the current study, RNAi screening of the hypoxic HIF-1α–NanoLuc reporter cells revealed that *RAF*, *MEK*, *ERK*, *MNK*, and *IGFR* act upstream of *HIF1A*, and that inhibition of *MAP2K1* decreased reporter activity the most (Figure 2A). Interestingly, the three identified MEK inhibitors— PD-184352, selumetinib, and trametinib — exhibited distinct activities against HIF-1 signaling and cancer cell proliferation. PD-184352 and trametinib inhibited HIF-1 activity in both HIF-1α–NanoLuc HCT116 and HRE–bla ME-180 assays; however, selmetinib reduced the levels of HIF-1α–NanoLuc but did not decrease HRE–bla transactivation activity (Table 1 and Table S8). Trametinib reduced HCT116 and ME-180 cell viability by half at submicromolar concentrations, whereas PD- 184352 and selumetinib required higher concentrations to achieve the same anti-proliferative effects on HCT116 cells (Table 1 and Table S2). Trametinib and selumetinib were approximately 60,000-fold and 150-fold more potent in blocking tube formation in an angiogenesis co-culture assay than in inhibiting HIF-1α–NanoLuc activity (Table 1). The gene expression profiling data showed that trametinib decreased expression levels of *HIF1A* and many hypoxia responsive genes in ME-180 cells (Figure 5B). In addition to MEK and HIF-1, there may have been other important targets and pathways that contribute to the sub-nanomolar anti-angiogenic activity of trametinib.

The cytotoxicity of PI3K pathway inhibitors in ME-180 cancer cells correlated well with their anti-HIF activity. Cells treated with inhibitors of PI3K and mTOR or transiently transfected with dominant negative *AKT* or *PI3K* have been reported to inhibit HIF-1α expression in human prostate cancer cell lines [24]. Our results show that the anti-HIF-1 activities of the PI3K pathway inhibitors measured by both HIF-1α–NanoLuc and HRE– bla assays correlated well (R^2^ > 0.75) with their effects on the viability of ME-180 cells (Table 1, Table S7, and Table S8). Moreover, siRNAs targeting *PIK3C* or *PIK3R* genes reduced HIF-1α–NanoLuc expression levels more than siRNAs targeting *AKT* or *FRAP* (Figure 2A). The results also suggested that PI3K pathway inhibitors modulate HIF-1 signaling by altering protein levels and transactivation activity of endogenous HIF-1α proteins. PI-103, the most potent novel HIF-1 inhibitor among the PI3K pathway inhibitors tested in this study, is a dual PI3K and mTOR inhibitor with anti-proliferative activity in leukemia [25], hepatocellular carcinoma [26], and glioma [27] cells. Unexpectedly, the mTOR inhibitor PP-242 and the two PI3K inhibitors PI- 103 and LY294002 exhibited a time-dependent increase in cytotoxicity to ME- 180 cells yet had no apparent anti-proliferative effects on HCT116 cells that had been continuously exposed to these compounds for three days. PI-103 and PP-242 exhibited similar potencies in their ability to inhibit HIF-1α–NanoLuc activity and block endothelial cell tube formation (Table 1). Consistent with the cell proliferation and angiogenesis assay data, PI-103 decreased the expression levels of all hypoxia-induced genes tested (Figure 5B).

The HIF-1α–NanoLuc assay identified several novel HIF-1 inhibitors including approved drugs such as mycophenolate mofetil and niclosamide. Mycophenolate mofetil, a prodrug of mycophenolic acid which was known to inhibit inosine monophosphate dehydrogenase (IMPDH), was used as an immunosuppressant in the clinic owing to its effects on purine synthesis [28]. Despite the fact that mycophenolate mofetil showed unsuccessful anti-tumor effects *in vivo*, this drug was reported to strongly inhibit VEGF secretion and angiogenesis in many cancer cell lines [29–31]. The anthelmintic drug niclosamide was recently discovered to have anti-cancer activities in colon cancer [32], lung cancer [33], breast cancer [34], and castration-resistant prostate cancer [35]. Niclosamide was reported to inhibit signal transducer and activator of transcription 3 (STAT3) [36], an upstream regulator of HIF-1 and VEGF signaling [37]. Our results show that both mycophenolate mofetil and niclosamide inhibit HIF-1α–NanoLuc accumulation and HRE–bla transactivation at similar potencies (Table 1). Mycophenolate mofetil selectively attenuated the viability of ME-180 cells and angiogenesis in the endothelial cell–mesenchymal stem cell co-cultures. Niclosamide was cytotoxic to HCT116 and ME-180 cells at low micromolar concentrations and anti-angiogenic at concentrations that were moderately cytotoxic. While both mycophenolate mofetil and niclosamide were able to reverse hypoxia-induced gene expression, mycophenolate mofetil was more similar to PI-103 and trametinib in modulating genes, such as hepatocyte nuclear factor 4 alpha (*HNF4A*) and nitric oxide synthase (*NOS3*) (Figure 5).

In summary, the present study systematically evaluated approved drugs, investigational drugs, and inhibition of druggable targets on HIF-1 signaling. Because HIF-1 regulates a wide variety of target genes required for angiogenesis, survival, metastasis, and glucose metabolism in cancer cells [21], it is important to explore whether existing anti-cancer agents could suppress tumor growth via HIF-1 inhibition, what approved drugs can be repurposed for anti-cancer drug development, and what upstream regulators will be most effective for modulating HIF-1 activity. As many of the potential regulators of HIF-1 have not known as the pharmacological modulators yet, there is a need to develop new therapeutic agents for these regulators. Due to the diverse, unexplored mechanisms of the HIF-1 inhibitors identified from this screen, it is necessary to further test various drug combinations in the same HIF-1 assays and follow-up studies for their *in vivo* anti-tumor activity.

## MATERIALS AND METHODS

### Reagents

Roswell Park Memorial Institute (RPMI) 1640 medium, Dulbecco's modified Eagle's medium (DMEM), Dulbecco's phosphate-buffered saline (DPBS), trypsin, 4-(2-hydroxyethyl)-1-piperazineethanesulfonic acid (HEPES), non-essential amino acids (NEAA), sodium pyruvate, blasticidin, penicillin/streptomycin, and CCF_4_ reagents were purchased from Life Technologies. Defined and dialyzed fetal bovine serum (FBS) was acquired from Thermo Fisher Scientific. ME-180 cell line, McCoy's 5A medium, and FBS were purchased from American Type Culture Collection (ATCC). Nano-Glo, CellTiter Blue, CellTiter Glo reagents were acquired from Promega. The NCATS Pharmaceutical Collection (NPC) library [8] was prepared as stock solutions in DMSO in 1536-well plates, where each plate corresponds to an indicated concentration of test compounds. G418 was purchased from Sigma-Aldrich. Azacitidine, AZD5363, bortezomib, camptothecin, enzastaurin, GDC-0994, GNF- 2, mitoxantrone, mycophenolate mofetil, niclosamide, OSI-906, PHT-427, PI-103, PP-242, selumetinib, TAK-632, trametinib, topotecan, volasertib, and YC-1 were purchased from SelleckChem. Cylcoheximide was purchased from Tocris Bioscience. The compound purity and identity were confirmed by quality control analysis at NCATS.

### Construction of X-MAN^®^ HIF-1α–NanoLuc reporter cell line

HCT116 cells, with a single allele of *HIF1A* endogenously tagged with a NanoLuc (Promega) reporter fusion, were generated using rAAV technologies, as previously described [38]. Briefly, a rAAV targeting vector with terminal viral inverted terminal repeat (ITR) sequences was synthesized using two regions of homology to the *HIF1A* endogenous locus and a floxed antibiotic selection marker. The 3′ homology region contained a linker sequence, followed by the NanoLuc open reading frame (ORF), immediately upstream of the endogenous STOP codon of *HIF1A* transcript ENST00000337138. After infection with the rAAV virus, antibiotic selection, and low density plate-out, clonal populations of targeted cells were selected and validated by PCR and Sanger sequencing to ensure populations were targeted at the correct locus and no off-target integrations of the targeting vector had occurred. Finally, Cre-recombinase was used to remove the selection cassette leaving a single LoxP sequence between the two regions of homology.

### HIF-1α–NanoLuc reporter gene assay, qHTS, and siRNA screening

X-MAN^®^ HIF-1α–NanoLuc cells were cultured in RPMI 1640 containing 10% defined FBS, 100 U/mL penicillin, 100 μg/mL streptomycin, and 300 μg/mL G418. Cells were seeded in white 96−, 384−, or 1536−well white solid bottom plates. After compound treatment under normoxic (20% O_2_) and hypoxic (1% O_2_) conditions, NanoLuc luciferase activity was measured using Nano-Glo reagent according manufacturer's instruction. For qHTS, cells at 1500 cells/well in 1536-well plates were incubated with test compounds at 37°C, 5% CO_2_, 1% O_2_ for 18 hours in a humidified CO_2_ incubator with variable oxygen control, followed by addition of Nano-Glo reagent or CellTiter-Glo cell viability assay reagent. The luminescence signals were collected on a ViewLux plate reader (Perkin Elmer). Analysis of compound concentration-response data and compound activity assignment was performed as previously described [5, 39]. For siRNA screening, cells in 384-well plates were reversely transfected with 20 nM siRNA duplexes from libraries of druggable targets (GE Dhamacon) using RNAiMAX (Life Technologies) and incubated for 42 hours prior to hypoxic conditions (1% O_2_) for six hours. The luciferase activity was quantified using Nano-Glo reagent and a FLUOstar OMEAGA plate reader (BMG Labtech). Screening analysis and quality control of the siRNA screening was conducted using KNIME (University of Konstanz) and Spotfire (TIBCO Software). The gene and pathway annotation was analyzed by Ingenuity Pathway Analysis software (QIAGEN).

### HRE-bla reporter gene assay

Cell culture of HRE–bla ME-180 cells (Cat. No. K1644, Life Technologies) and HRE–bla assay were conducted according to a reported protocol [6]. Briefly, HRE–bla cells were seeded in 1536-well black clear bottom plates (Greiner Bio One) and treated with test compounds at 37°C, 5% CO_2_, 1% O_2_ for 18 hours, followed by addition of a beta-lactamase substrate CCF_4_ and CellTiter-Glo reagent. The fluorescence signals (460/25 nm and 530/20 nm) of CCF_4_ and luminescence signals of CellTiter-Glo were acquired on an Envision and a ViewLux plate readers (Perkin Elmer), respectively.

### Cancer cell proliferation assay

HCT116 and ME-180 cells (ATCC) were cultured according to manufacturer's instructions. Cells were seeded in 1536-well plates and treated with test compounds at 11 concentrations for 24, 48, and 72 hours at 37°C, 5% CO_2_. Cell proliferation was quantified as relative luminescence unit (RLU) values using CellTiter-Glo viability assay reagent on a ViewLux plate reader (Perkin Elmer).

### *In vitro* angiogenesis co-culture assay

The angiogenesis co-culture assay was conducted using Angio-Ready Angiogenesis Assay (Cat. No. ACS-2001, ATCC) according to manufacturer instructions. Cells were seeded in a 96-well clear bottom plate (Corning) for five hours and exposed to test compounds for three days at 37°C, 5% CO_2_. Tube formation and cell viability of each well were acquired on an ArrayScan VTI reader (Thermo Fisher Scientific) with a 5X objective and 488ex/530em filters to image the GFP-expressing tubular structures and on a ViewLux plate reader (PerkinElmer) using CellTiter-Glo reagent, respectively.

### RNA isolation, cDNA synthesis, and real-time qPCR

ME-180 cells were treated for 18 hours at 37°C, 5% CO_2_, 1% O_2_ with DMSO, mycophenolate mofetil, niclosamide, or trametinib at their IC_50_ concentrations established by the HIF-1α-NanoLuc assay. Total RNA of each sample was isolated from cell lysates using RNeasy Mini kit (QIAGEN) and converted to cDNA using RT2 First Strand kit (QIAGEN). Gene expression of each cDNA sample was amplified using SYBR Green and RT^2^ Profiler PCR Array Human Hypoxia Signaling Pathway kits (QIAGEN), measured on ViiA7 Real-Time PCR System (Applied Biosystems), and analyzed using GeneGlobe software (QIAGEN) and beta-2-microglobulin (B2M) as an endogenous control.

## SUPPLEMENTARY MATERIALS TABLES AND FIGURES


