# Supplementary material for: Identification of approved and investigational drugs that inhibit hypoxia-inducible factor-1 signaling
Source: Oncotarget. 2016 Jan 23;7(7):8172–83. doi: 10.18632/oncotarget.6995 (PMC4884984; doi:10.18632/oncotarget.6995)
Supplement: Supplementary file 1 [file oncotarget-07-8172-s001.pdf]

Identification of approved and investigational drugs that inhibit hypoxia-inducible factor-1 signaling

Supplementary Materials

Supplementary Table S1: Known HIF-1 inhibitors and their analogs identified in the primary screen using the HIF-1α–NanoLuc assay

Compound Name (CASN)	IC_{50} (μM)	Efficacy (%)	Structure Class	Description	Known HIF-1 inhibitor?
Actinomycin D (50-76-0)	0.04	99	Phenoxazine	Anti-cancer drug. DNA binder and transcription inhibitor.	Yes [1]
Berberine (633-65-8)	0.94	116	Alkaloid	Anti-parasitic, anti-fungal, and anti-diarrheal drug.	Yes [1]
Celecoxib (169590-42-5)	10.59	43	NSAID	Anti-inflammatory drug. COX2 inhibitor.	Yes [2]
Cilnidipine (132203-70-4)	10.59	63	Dihydropyridine	Anti-hypertension drug. Calcium channel blocker.	Yes [3]
Cycloheximide (66-81-9)	0.24	92	N/A	Translation inhibitor.	Yes [1]
Cyclosporine (59865-13-3)	11.88	87	Cyclic peptide	Immunosuppressant drug.	Yes [1]
Daunorubicin (20830-81-3)	0.75	51	Anthracycline	Anti-cancer drug. DNA intercalator and topoisomerase inhibitor.	Yes [4]
Deslanoside (17598-65-1)	0.30	115	Cardiac glycoside	Drug to treat congestive heart failure.	No
Digitoxin (71-63-6)	0.03	124	Cardiac glycoside	Drug to treat congestive heart failure.	Yes [5]
Disulfiram (97-77-8)	0.03	89	Carbamate	Drug to treat alcohol dependence. ALDH inhibitor.	Yes [6]
Emetine (316-42-7)	0.19	136	Alkaloid	Anti-protozoal and drug to induce vomiting.	Yes [7]
Erlotinib (183321-74-6)	2.66	33	Quinazoline	Anti-cancer drug. EGFR inhibitor.	Yes [2]
Epirubicin (56390-09-1)	0.30	108	Anthracycline	Anti-cancer drug. DNA intercalator.	No
Flavopiridol (146426-40-6)	1.06	76	Flavonoid alkaloid	Anti-cancer drug. CDK inhibitor.	Yes [8]
Gefitinib (184475-35-2)	0.19	86	Quinazoline	Anti-cancer drug. EGFR inhibitor.	Yes [2]
Genistein (446-72-0)	8.41	65	Isoflavone	Supplement.	Yes [1]
Gimatecan (292618-32-7)	0.60	108	Quinoline alkaloid	Anti-cancer drug. Topoisomerase I inhibitor.	No
Idarubicin (57852-57-0)	0.75	61	Anthracycline	Anti-cancer drug. DNA intercalator.	No

Isradipine (75695-93-1)	11.88	111	Dihydropyridine	Anti-hypertension drug. Calcium channel blocker.	No
Lercanidipine (132866-11-6)	11.88	63	Dihydropyridine	Anti-hypertension drug. Calcium channel blocker.	No
Manidipine (89226-50-6)	9.44	86	Dihydropyridine	Anti-hypertension drug. Calcium channel blocker.	No
Mitoxantrone (70476-82-3)	2.38	80	Anthracenedione	Drug to treat multiple sclerosis.	Yes [9]
Nelfinavir (159989-65-8)	21.13	120	Benamide	Anti-HIV drug. HIF-1 protease inhibitor.	Yes [10]
Nemorubicin (108852-90-0)	0.08	111	Anthracycline	Anti-cancer drug. DNA intercalator.	No
Nicardipine (54527-84-3)	11.88	147	Dihydropyridine	Anti-hypertension drug. Calcium channel blocker.	No
Nimodipine (66085-59-4)	14.96	132	Dihydropyridine	Anti-hypertension drug. Calcium channel blocker.	No
Nitrendipine (39562-70-4)	14.96	157	Dihydropyridine	Anti-hypertension drug. Calcium channel blocker.	No
Ouabain (11018-89-6)	0.08	130	Cardiac glycoside	Drug to treat congestive heart failure.	Yes [5]
Pirarubicin (72496-41-4)	11.88	97	Anthracycline	Anti-cancer drug. DNA intercalator.	No
Proscillaridin A (466-06-8)	0.01	101	Cardiac glycoside	Drug to treat congestive heart failure and cardiac arrhythmia.	Yes [5]
Rotenone (83-79-4)	0.19	91	Rotenone	Insecticide. Inhibitor of mitochondrial election transport.	Yes [1]
Sorafenib (284461-73-0)	13.33	156	Diarylurea	Anti-cancer drug. RAF inhibitor.	Yes [11]
Topotecan (123948-87-8)	6.68	98	Quinoline alkaloid	Anti-cancer drug. Topoisomerase I inhibitor.	Yes [2]
Vatanidipine (116308-55-5)	1.19	103	Dihydropyridine	Anti-hypertension drug. Calcium channel blocker.	No
Vorinostat (149647-78-9)	1.50	51	Hydroxyxamic acid	Anti-cancer drug. HDAC inhibitor.	Yes [2]

ALDH: Acetaldehyde dehydrogenase. CDK: Cylin-dependent kinase. EGFR: Epidermal growth factor receptor. HDAC: Histone deacetylase. HIV: human immunodeficiency virus. NSAID: Non-steroidal anti-inflammatory drug

Supplementary Table S2: Effects of small molecular inhibitors and siRNA oligos on HIF-1α–NanoLuc activity

Target	Pathway inhibitors (primary screen) HIF-1α–NanoLuc Activity IC_{50} (Efficacy)	Pathway inhibitors (validation) HIF-1α–NanoLuc Activity IC_{50} (Efficacy)	siRNA oligos HIF-1α–NanoLuc Activity* (Viability Cell Number)
Akt/PKB	MK-2206: 0.42 μM (45%)	AZD5363: 1.50 ± 0.21 μM (71 ± 5%)	*AKT1*: 45 ± 2% (88%)<br>*AKT2*: 69 ± 5% (102%)<br>*AKT3*: 19 ± 0% (58%)
BCR/ ABL	Bosutinib: 11.88 μM (58%)<br>Dasatinib: 0.94 μM (82%)<br>Imatinib: 33.49 μM (28%)	Dasatinib: 23.58 ± 8.59 μM (46 ± 3%)	*BCR*: 11 ± 1% (62%)<br>*ABL1*: 141 ± 17% (110%)<br>*ABL2*: 71 ± 10% (54%)
EGFR	Canertinib: 2.37 μM (50%)<br>Erlotinib: 2.66 μM (33%)<br>Gefitnib: 18.83 μM (57%)<br>Lapatinib: Inactive<br>Tandutinib: Inactive	Canertinib: 24.84 ± 10.72 μM (88 ± 20%)<br>Erlotinib: 6.64 ± 2.42 μM (35 ± 6%)<br>Gefitinib: 19.41 ± 1.31 μM (31 ± 3%)<br>Lapatinib: 20.17 ± 1.31 μM (50 ± 6%)	*EGFR*: 10 ± 1% (50%)
ERK	N/A	GDC-0994: 6.11 ± 1.64 μM (63 ± 7%)	*MAPK1*: 52 ± 10% (61%)<br>*MAPK3*: 52 ± 6% (84%)
IGF and Insulin receptor	Picropodphyllin: 0.53 μM (159%*)	Picropodphyllin: 0.53 μM (159%*)	*IGF1R*: 57 ± 5% (87%)<br>*IGF2R*: 60 ± 5% (81%)<br>*INSR*: 92 ± 2% (73%)<br>*INSRR*: 73 ± 1% (87%)
HDAC	Quercetine: 0.84 μM (44%)<br>Vorinostat: 1.50 μM (51%)	Trichostain A: 0.67 ± 0.26 μM (55 ± 4%)	N/A
MEK	PD 184352: 0.84 μM (64%)<br>Selumetinib: 0.04 μM (62%)	PD 184352: 13.74 ± 0.93 μM (99 ± 2%)<br>Selumetinib: 1.49 ± 1.30 μM (48 ± 10%)<br>Trametinib: 2.98 ± 4.64 nM (69 ± 5%)	*MAP2K1*: 21 ± 3% (74%)<br>*MAP2K2*: 89 ± 8% (103%)
MNK	N/A	N/A	*MKNK1*: 117 ± 6% (100%)<br>*MKNK2*: 56 ± 2% (79%)
mTOR	PP-242: 0.05 μM (34%)	PP-242: 0.45 ± 0.20 μM (88 ± 2%)	*FRAP1*: 43 ± 11% (78%)
p70 S6K	N/A	N/A	*RPS6KB1*: 28 ± 1% (69%)<br>*RPS6KB2*: 52 ± 2% (97%)
PDK	N/A	N/A	*PDPK1*: 53 ± 2% (91%)
PI3K	N/A	LY-294002: 14.58 ± 3.54 μM (50 ± 9%)<br>PI-103: 2.77 ± 0.04 μM (80 ± 3%)<br>Wortmannin: 0.99 ± 0.22 μM (71 ± 4%)	*PIK3R1*: 72 ± 5% (110%)<br>*PIK3R2*: 41 ± 0% (72%)<br>*PIK3R3*: 108 ± 19% (109%)<br>*PIK3R4*: 15 ± 1% (92%)<br>*PIK3C2A*: 4 ± 0% (33%)<br>*PIK3C2B*: 73 ± 3% (77%)<br>*PIK3C2G*: 13 ± 1% (73%)<br>*PIK3C3*: 49 ± 4% (75%)<br>*PIK3CA*: 42 ± 1% (88%)<br>PIK3CB: 95 ± 3% (95%)<br>PIK3CD: 113 ± 8% (79%)<br>*PIK3CG*: 44 ± 6% (91%)

PKC	Enzastaurin: 14.96 μM (39%)<br>Quercetine: 0.84 μM (44%)<br>Tamoxifen: 29.85 μM (96%)	Enzastaurin: 2.06 ± 3.30 μM (67 ± 6%)<br>Quercetine: 37.22 ± 0.00 μM (37 ± 1%)<br>Tamoxifen: 30.73 ± 5.74 μM (107 ± 6%)	*PRKCA*: 46 ± 3% (73%)<br>*PRKCB1*: 32 ± 1% (69%)<br>*PRKCD*: 31 ± 4% (68%)<br>*PRKCE*: 53 ± 2% (80%)<br>PRKCG: 76 ± 2% (82%)<br>*PRKCH*: 26 ± 5% (55%)<br>*PRKCI*: 38 ± 2% (84%)<br>*PRKCL1*: 103 ± 13% (97%)<br>*PRKCL2*: 63 ± 1% (75%)<br>*PRKCM*: 23 ± 3% (74%)<br>*PRKCN*: 23 ± 4% (61%)<br>*PRKCQ*: 86 ± 8% (81%)<br>*PRKCSH*: 69 ± 2% (81%)<br>*PRKCZ*: 36 ± 4% (81%)
PLK	N/A	Volasertib: 9.57 ± 5.60 μM (78 ± 14%)	*PLK1*: 7 ± 1% (28%)<br>*PLK2*: 112 ± 13% (78%)<br>*PLK3*: 36 ± 1% (83%)<br>*PLK4*: 18 ± 1% (68%)
RAF	Sorafenib: 1.50 μM (46%)	Dabrafenib: 0.47 ± 0.24 μM (26 ± 13%)<br>Sorafenib: 23.59 ± 2.71 μM (100 ± 6%)<br>TAK-632: 1.41 ± 0.23 μM (85 ± 2%)<br>Vemurafenib: 13.59 ± 6.38 μM (66 ± 6%)	*ARAF1*: 88 ± 4% (72%)<br>*BRAF*: 27 ± 3% (64%)<br>*RAF1*: 11 ± 1% (78%)
SIRT	N/A	AGK2: 19.50 ± 2.49 μM (107± 9%)<br>Cambinol: Inactive<br>EX-527: Inactive<br>Quercetine: 37.22 ± 0.00 μM (37 ± 1%)<br>Resveratrol: 41.10 ± 8.51 μM (43 ± 12%)<br>Suramin: 24.54 ± 3.13 μM (41 ± 7%)	*SIRT2*: 45 ± 13% (59%)<br>*SIRT3*: 62 ± 9% (64%)<br>*SIRT4*: 156 ± 52% (65%)<br>*SIRT5*: 194 ± 37% (90%)<br>*SIRT6*: 53 ± 15% (52%)<br>*SIRT7*: 118 ± 23% (88%)
TOPO	Daunorubicin: 0.75 μM (50%)<br>Gimatecan: 0.60 μM (108%)<br>Mitoxantrone: 2.37 μM (80%)<br>Quercetine: 0.84 μM (44%)<br>Topotecan: 6.68 μM (98%)	Camptothecin: 0.57 ± 0.04 μM (102 ± 5%)<br>Daunorubicin: 1.96 ± 0.35 μM (98 ± 8%)<br>Mitoxantrone: 7.65 ± 2.13 μM (110 ± 5%)<br>Quercetine: 37.22 ± 0.00 μM (37 ± 1%)<br>Topotecan: 2.10 ± 0.24 μM (99 ± 6%)	N/A
VEGFR	Axitinib: Inactive<br>Sorafenib: 1.50 μM (46%)<br>SU-5416: 23.71 μM (67%)<br>Sunitinib: 1.68 μM (60%)	Sorafenib: 23.59 ± 2.71 μM (100 ± 6%)<br>SU-5416: 12.23 ± 7.55 μM (57 ± 22%)<br>Sunitinib: 11.34 ± 3.71 μM (80 ± 15%)	*FLT1*: 172 ± 5% (124%)<br>*FLT3*: 52 ± 1% (83%)<br>*FLT4*: 10 ± 3% (51%)
VHL	N/A	N/A	*VHL*: 19 ± 4% (35%)

*The HIF-1α-NanoLuc intensity values of each siRNA experiment were normalized to a positive transfection control (full inhibition; 0% HIF-1α-NanoLuc activity) and a non-targeting reference (no activation/inhibition; 100% HIF-1α-NanoLuc activity).

IGF: Insulin-like growth factor. IGFR: Insulin-like growth factor receptor. INSR: Insulin receptor. INSRR: Insulin receptor-related receptor. MAP2K: Mitogen-activated protein kinase kinase. MAPK: Mitogen-activated protein kinase. MNK or MKNK: MAPK-interacting serine/threonine-protein kinase. PI3K: Phosphoinositide 3-kinase. Akt/PKB: Protein kinase B. mTOR: Mammalian target of rapamycin. PDK or PDPK: Proline-directed protein kinase. VHL: Von Hippel-Lindau tumor suppressor. SIRT: Sirtuin. PLK: Polo-like kinase.

Supplementary Table S3: Top 10 canonical pathways identified by siRNA screen using the HIF-1α–NanoLuc assay

Rank	Canonical Pathways	Score -log(*p*-value)	Ratio	Targets
1	Salvage pathways of primidine ribonucleotides	78.80	79/94 (79%)	*MAP2K4, MAPK1, SGK1, CSNK1A1, NME2, LIMK2, LIMK1, NME6, PAK1, AK1, CDK8, MAP2K2, PIM1, MAPK3, PRPF4B, PRKAA1, HIPK1, AK7, MAP3K9, CDK18, GRK4, NME4, AKT2, DAPK1, PRKCQ, NME5, CDK7, CSNK1D, CDK6, GRK5, PKN1, NME3, ARAF, PRKCD, GRK6, UCK1, PRKCH, MAP2K3, PAK7, MAPK7, NEK2, MAK, CDK2, MAP2K6, NME1, DMPK, CDK4, TTK, NME7, IRAK1, BRAF, PRKX, CDK5, PRKAA2, PRKCE, CMPK1, MAP2K1, MAP3K6, MAPK8, MAPK6, MAPK9, PLK1, CDK1, AK5, PAK3, PAK2, MAP3K8, AK4, POMK, UCK2, EIF2AK2, UCKL1, ACVR2A, DYRK1A*
2	Pyridoxal 5′-phosphate salvage pathway	74.00	60/64 (94%)	*MAP2K4, MAPK1, SGK1, CSNK1A1, LIMK2, LIMK1, PAK1, CDK8, MAP2K2, PIM1, MAPK3, PRPF4B, PRKAA1, HIPK1, MAP3K9, CDK18, GRK4, AKT2, DAPK1, PRKCQ, CDK7, CDK6, CSNK1D, GRK5, PKN1, ARAF, PRKCD, GRK6, PRKCH, PAK7, MAP2K3, MAPK7, NEK2, MAK, CDK2, MAP2K6, DMPK, CDK4, TTK, IRAK1, BRAF, PRKX, CDK5, PRKAA2, PRKCE, MAP2K1, PDXK, MAP3K6, MAPK8, MAPK6, MAPK9, PLK1, CDK1, PAK3, PAK2, MAP3K8, POMK, EIF2AK2, ACVR2A, DYRK1A*
3	NFκB signaling	57.20	65/107 (61%)	*MAP2K4, MAP3K15, RAF1, MAP3K11, MAPK1, PIK3R1, PDPK1, MAP3K4, PIK3R4, ROCK2, IKBKB, MAP3K10, IKBKG, MAP2K2, MAPK3, PIK3CG, MAP3K7, ATM, MAP3K2, PIK3C2B, MAP3K14, MAP3K9, RPS6KB1, AKT2, MAP3K13, RAC1, MAPK12, TRAF6, PIK3R3, MAP3K12, RPS6KA6, PRKCD, MAPK10, PIK3CD, RPS6KA1, MAPK7, RELA, PIK3CA, RPS6KA3, PIK3R5, TRIO, MAP3K5, PRKCZ, AKT1, PIK3C3, NTRK1, RPS6KB2, AKT3, RPS6KA2, CHUK, PIK3R2, MAP2K1, MAP3K6, PIK3C2A, MAP3K1, MAPK8, PIK3C2G, MAPK9, IKBKE, RPS6KA5, ROCK1, MAP3K8, PIK3CB, RPS6KA4, MAP3K3*
4	NGF signaling	52.70	79/173 (46%)	*PRKACB, RAF1, TGFBR1, TGFBR3, PIK3R1, BMPR1B, PIK3R4, TGFBR2, FGFR3, IKBKB, IKBKG, LCK, FGFR4, PIK3CG, CSNK2A1, MAP3K7, GSK3B, TAB1, PDGFRB, ATM MAP3K14, PIK3C2B, AKT2, PRKCQ, FGFR1, FGFR2, TBK1, DDR1, PIK3R3, TRAF6, ARAF, PRKACG, ZAP70, PRKACA, PIK3CD, INSR, FGFRL1, MAP2K6, RELA, PIK3CA, PIK3R5, BMPR2, TNFAIP3, PRKCZ, IRAK1, BRAF, AKT1, BMPR1A, PIK3C3, NTRK1, PDGFRA, IGF1R, AKT3, PIK3R2, CSNK2B, CHUK, EGFR, MAP2K7, FLT1, PIK3C2A, MAP3K1, FLT4, MAPK8, PIK3C2G, IRAK3, IGF2R, CSNK2A2, TLR4, NTRK2, RIPK1, NTRK3, MAP3K8, PIK3CB, EIF2AK2, KDR, MAP4K4, MAP3K3, IRAK4, PRKCB*
5	GNRH signaling	52.60	67/129 (52%)	*MAP2K4, PRKACB, MAP3K15, RAF1, MAP3K11, MAPK1, MAP3K4, MAPK13, PTK2, MAP3K10, PAK1, CAMK2D, CAMK2A, MAP2K2, MAPK3, MAP3K7, PRKD3, PRKD1, MAP3K2, MAP3K14, MAP3K9, PRKCQ, MAP3K13, RAC1, MAPK12, MAP3K12, PRKCD, PRKACG, PRKACA, MAPK10, PRKCH, MAP2K3, PAK7, MAPK7, CAMK2G, MAP2K6, RELA, MAP3K5, MAPK11, PRKCZ, PRKAG1, PRKAR1B, PRKCE, MAP2K1, EGFR, PRKCA, CAMK2B, SRC, MAP2K7, PAK4, PAK6, MAP3K6, MAP3K1, PRKAR2A, MAPK8, MAPK9, PRKCG, PRKAR2B, MAPK14, PRKCI, PAK3, PAK2, PRKAG2, MAP3K8, MAP3K3, PRKAR1A, PRKCB*

6	RANK signaling in osteoclasts	52.00	0.65	*MAP2K4, RAF1, MAP3K15, MAP3K11, MAPK1, PIK3R1, MAPK13, MAP3K4, PIK3R4, IKBKB, MAP3K10, IKBKG, MAP2K2, MAPK3, PIK3CG, MAP3K7, ATM, MAP3K2, PIK3C2B, MAP3K14, MAP3K9, AKT2, MAP3K13, MAPK12, TRAF6, PIK3R3, MAP3K12, MAPK10, PIK3CD, MAP2K6, RELA, PIK3CA, CAMK4, PTK2B, PIK3R5, MAP3K5, MAPK11, AKT1, PIK3C3, AKT3, PIK3R2, CHUK, MAP2K1, SRC, MAP2K7, PIK3C2A, MAP3K6, MAP3K1, PIK3C2G, MAPK8, MAPK9, IKBKE, CALM1, MAPK14, MAP3K8, PIK3CB, MAP3K3*
7	HGF signaling	51.80	0.58	*MAP2K4, MAP3K15, RAF1, MAP3K11, MAPK1, PIK3R1, MAP3K4, PIK3R4, PTK2, MAP3K10, PAK1, MAP2K2, MAPK3, PIK3CG, MAP3K7, PRKD3, PRKD1, ATM, MAP3K2, PIK3C2B, MAP3K14, MAP3K9, AKT2, PRKCQ, CRKL, MAP3K13, RAC1, MAPK12, MET, PIK3R3, MAP3K12, PRKCD, MAPK10, PIK3CD, PRKCH, CDK2, PIK3CA, PIK3R5, MAP3K5, PRKCZ, AKT1, PIK3C3, AKT3, PRKCE, PIK3R2, MAP2K1, PRKCA, MAP2K7, MAP3K6, PIK3C2A, MAP3K1, PIK3C2G, MAPK8, MAPK9, PRKCG, PRKCI, CDKN1A, MAP3K8, PIK3CB, MAP3K3, PRKCB*
8	ErbB signaling	50.60	0.65	*MAP2K4, RAF1, MAPK1, PIK3R1, PDPK1, MAPK13, PIK3R4, PAK1, MAP2K2, PIK3CG, MAPK3, GSK3B, PRKD3, PRKD1, ATM, PIK3C2B, RPS6KB1, PRKCQ, MAPK12, PIK3R3, PRKCD, MAPK10, PRKCH, PIK3CD, PAK7, MAP2K3, MAP2K6, PIK3CA, PIK3R5, MAPK11, PRKCZ, MTOR, AKT1, ERBB4, PIK3C3, PRKCE, ERBB2, PIK3R2, MAP2K1, EGFR, PRKCA, PAK4, PIK3C2A, PAK6, MAPK8, PIK3C2G, MAPK9, ERBB3, PRKCG, MAPK14, PRKCI, PAK3, NRG3, PAK2, PIK3CB, PRKCB*
9	PTEN signaling	49.90	0.53	*RAF1, TGFBR1, MAPK1, TGFBR3, PIK3R1, PDPK1 GSK3A, BMPR1B, TGFBR2, FGFR3, PTK2, IKBKB, IKBKG, MAP2K2, MAPK3, FGFR4, PIK3CG, CSNK2A1, GSK3B, PDGFRB, RPS6KB1, AKT2, FGFR1, RAC1, FGFR2, DDR1, PIK3R3, PIK3CD, FGFRL1, INSR, MAGI3, MAST2, RELA, PIK3CA, ILK, PIK3R5, BMPR2, PRKCZ, AKT1, BMPR1A, NTRK1, PDGFRA, IGF1R, RPS6KB2, AKT3, PIK3R2, CHUK, CSNK2B, MAP2K1, EGFR, FLT1, FLT4, IKBKE, IGF2R, CSNK2A2, MAGI1, NTRK2, NTRK3, CDKN1A, PIK3CB, CDKN1B, KDR, MAGI2*
10	Molecular mechanisms of cancer	49.80	0.28	*RAF1, TGFBR1, SUV39H1, PIK3R1, CDKN2C, GSK3A, BMPR1B, MAPK13, PAK1, CAMK2A, PIK3CG, HIPK2, PRKD3, PRKD1, ATM, PIK3C2B, AKT2, PRKCQ, STK36, CDK7, PRKACA, PIK3CD, PAK7, MAP2K3, CAMK2G, MAP2K6, FYN, PIK3CA, CDK4, BMPR2, JAK2, MAP3K5, PRKCZ, CHEK1, AKT1, PIK3C3, PRKCE, PIK3R2, CAMK2B, PRKDC, PIK3C2G, MAPK8, SIN3A, PRKCG, CDKN2D, PAK3, CDKN1A, JAK3, PRKCB, PRKACB, MAP2K4, JAK1, MAPK1, PIK3R4, TGFBR2, PTK2, CAMK2D, MAP2K2, MAPK3, MAP3K7, GSK3B, TAB1, TYK2, RAC1, CDK6, AURKA, MAPK12, PIK3R3, PRKCD, PRKACG, MAPK10, PRKCH, CDK2, RELA, PIK3R5, ABL1, CDKN2B, MAPK11, PRKAG1, BRAF, NLK, CDK5, BMPR1A, PRKAR1B, AKT3, MAP2K1, CHEK2, PRKCA, SRC, PAK4, PIK3C2A, PAK6, PRKAR2A, MAPK9, PRKAR2B, PRKCI, MAPK14, PAK2, PRKAG2, ATR, PIK3CB, CDKN1B, PRKAR1A*

Supplementary Table S4: Top 10 disease and biological function networks identified by siRNA screen using the HIF-1α–NanoLuc assay

Rank	Top Disease and Functions	Score -log (*p*-value)	Focus Molecules	Molecules in Network
1	RNA post-transcriptional modification, post-translational modification, cancer	51	34	*CDK11B, DSTYK, DYRK1A, MAPK3, Mi2, MPND, MST4, PAN2, PCBP1, PIM3, PNKP, PRKDC, PRPF8, PRPF4B, RYK, SF3B2, SMARCAD1, SMG1, SRPK1, SRPK2, SRPK3, SRSF1, SRSF2, SRSF3, STK24, STK25, TMPRSS2, TRIM23, TRIM27, TRIM32, UBE2K, URM1, USP30, USP36, ZRSR2*
2	Cancer, gastrointestinal disease, post-translational modification	45	32	*BRSK1, BRSK2, DUB, INSRR, JOSD1, MARK1, MARK2, MARK3, MLKL, NFkB, NUAK2, OTUB2, peptidase, PKN3, RIOK3, RIPK4, STK10, STRADA, STRADB, TAOK1, UHMK1, USP21, USP26, USP28, USP29, USP31, USP34, USP35, USP38, USP40, USP43, USP51, USP27X, USP9Y, USPL1*
3	Cancer, organismal development, cell death and survival	40	30	*ADCK5, caspase, CDK4, CDK17, CDK11A, DLG4, GCK, GNRH, GRK5, HIPK1, Histone H4, MAGI2, MAPK7, MAPK8, MAPK9, MKK6/7, Nos, NRK, PAK1, PCDH15, PCLO, PDK2, PRKCE, SIAH1, SMYD5, SRP72, STK3, STK4, SUV420H2, TBCK, THNSL1, TRIM33, UBE2I, USP47, VRK1*
4	Post-translational modification, protein degradation, protein synthesis	40	30	*20s proteasome, α-catenin, BAP, BRCC, Calmodulin, CLK, DAPK, F-actin, PKN2, PSMA3, PSMD14, Spectrin, SPTA1, STK33, UCHL1, UCHL5, UFD1L, USP1, USP5, USP6, USP7, USP8, USP11, USP12, USP13, USP14, USP15, USP19, USP20, USP22, USP32, USP39, USP46, USP9X, YOD1*
5	Carbohydrate metabolism, lipid metabolism, small molecule biochemistry	36	28	*15-LOX, AGK, CLK1, CLK4, COL4A3BP, CSNK1G2, EGFR, GAD, GAK, Grb2-Shc1-Sos, MAST1, PDK3, Phosphatidylinositol4,5 kinase, PI4K, PI4K2A, PI4K2B, PI4KA, PI4KB, PI4P5K, PIKFYVE, PIP4K2A, PIP4K2B, PIP4K2C, PIP5K1A, PIP5K1B, PIP5K1C, PIP5KL1, PKC alpha/beta, PXK, RIOK1, ROR1, ROS1, STAMBP, STAMBPL1, UBL5*
6	Post-translational modification, cell-to-cell signaling and interaction, molecular transport	35	28	*7S NGF, AATK, AKT2, ARAF, CDC42BPA, Cofilin, CSNK1A1, ENaC, FXN, GRK4, Limk, LIMK1, LIMK2, MAP2K6, MAP4K2, MTORC2, OXSR1, P38 MAPK, PAK2, PAK3, PAK4, PASK, PDK1, PKN1, PRKCH, PRKCQ, SGK1, STK39, TESK1, tubulin, WNK1, WNK2, WNK3, WNK4, ZMYND8*
7	Post-translational modification, cancer, gastrointestinal disease	34	27	*AMHR2,BMPR2,BMPR1A,BMPR1B,Collagen type III,Collagen type IV, DDR1, FGFR1, FGFR2, FGFR3, FGFR4, FGFRL1, FLT1, FLT4, IGF1R, IGF2R, KDR, Laminin1, NME2, NME3, NTRK1, Patched, PDGF Ab, PDGFRA, PDGFRB, PKC (α,ε,θ), PLC gamma, SIRT6, SLC16A2, STK35, TGFBR1, TGFBR2, Type I /Type II receptor, USP10, USP50*

8	Post-translational modification, cellular development, cellular growth and proliferation	34	27	*ABL1, BRD2, CALM1, CAMKV, CDK10, CK2, CSNK2A1, CSNK2A2, cytochrome C, EXOSC10, HIPK4, Histone H3, IL1, IL12, INSR, MTOR, NMDA Receptor, PCDHA12, PRKACA, PRKACB, RAB37, RAF1, RNA polymerase II, SIN3A, SIRT7, SMARCA5, TET1, TET2, tubulin, ULK2, USP3, USP24, USP44, USP49, ZNF781*
9	Post-translational modification, cell cycle, DNA replication, recombination, and repair	33	28	*APC, AURK, AURKA, AURKB, AURKC, BMP2K, BUB1, BUB1B, CDC25, CHEK2, CMPK1, DBF4, DTYMK, GSG2, MELK, NAGK, NEK3, NEK6, NEK7, NEK9, PDE4, PKDCC, PLK, PLK1, PLK2, PLK3, PLK4, SNRK, STK16, UCK1, UCK2, UCKL1, uridine kinase, VEGF, XIRP2*
10	Post-translational modification, cancer, gastrointestinal disease	30	25	*ACVR1C, CAMK2N1, CKS2, EIF2AK4, Eph Receptor, EPHA7, EPHA10, ERK, ETS, GK, KSR1, KSR2, MAGI, MAGI3, MAPK4, MAPK12, MAPK15, MAPK kinase, MAPKAPK3, MAPKAPK2/3, MATK, MKNK1, MKNK2, MLK, PDK4, PEAK1, PRKG2, RhoGap, SGK223, SMAD1/5, SULF1, TMSB4, TRIB1, VRK2, ZNF267*

Supplementary Table S5: Top 20 genes up-regulated by siRNA oligos, measured by HIF-1α–NanoLuc assay

Rank	GenBank ID	Symbol	Gene Name	Activation %* HIF-1α-NanoLuc (*n* = 3)	Cell Viability % (*n* =1)	Networks in IPA
1	NM_012241	*SIRT5*	Sirtuin 5	194 ± 37	90	N/A
2	NM_032435	*KIAA1804*	Mixed lineage kinase 4	188 ± 16	115	13
3	NM_020730	*DLG3*	Discs, large homolog 3 (Drosophila)	180 ± 16	117	12
4	NM_002019	*FLT1*	Fms-related tyrosine kinase 1	172 ± 5	124	7
5	NM_014975	*MAST1*	Microtubule associated serine/ threonine kinase 1	167 ± 17	114	5
6	NM_007170	*TESK2*	Testis-specific kinase 2	166 ± 16	131	N/A
7	NM_001715	*BLK*	B lymphoid tyrosine kinase	163 ± 10	97	11
8	NM_006282	*STK4*	Serine/threonine kinase 4	163 ± 24	102	3
9	NM_001005915	*ERBB3*	v-erb-b2 avian erythroblastic leukemia viral oncogene homolog 3	161 ± 5	96	15
10	NM_182691	*SRPK2*	SRSF protein kinase 2	160 ± 5	97	1
11	NM_001006943	*EPHA8*	EPH receptor A8	159 ± 23	68	N/A
12	NM_005009	*NME4*	NME/NM23 nucleoside diphosphate kinase 4	158 ± 4	110	15
13	NM_012240	*SIRT4*	Sirtuin 4	156 ± 51	65	N/A
14	NM_020903	*USP29*	Ubiquitin specific peptidase 29	153 ± 32	96	2
15	NM_138316	*PANK1*	Pantothenate kinase 1	153 ± 38	63	15
16	NM_001656	*TRIM23*	Tripartite motif containing 23	153 ± 12	76	1
17	NM_020630	*RET*	Ret proto-oncogene	152 ± 8	101	11
18	NM_004614	*TK2*	Thymidine kinase 2, mitochondrial	147 ± 11	97	N/A
19	NM_004936	*CDKN2B*	Cyclin-dependent kinase inhibitor 2B	143 ± 8	100	N/A
20	NM_004563	*PCK2*	Phosphoenolpyruvate carboxykinase	141 ± 31	105	N/A

*The HIF-1α-NanoLuc intensity values of each siRNA experiment were normalized to a positive transfection control (full inhibition; 0% HIF-1α-NanoLuc activity) and a non-targeting reference (no activation/inhibition; 100% HIF-1α-NanoLuc activity).

Supplementary Table S6: Top 20 genes down-regulated by siRNA oligos, measured by HIF-1α–NanoLuc assay

Rank	GenBank ID	Symbol	Gene Name	Activation %* HIF-1α-NanoLuc (*n* = 3)	Cell Number % (*n* =1)	Networks in IPA
1	NM_002645	*PIK3C2A*	phosphatidylinositol-4-phosphate 3-kinase, catalytic subunit type 2 alpha	4 ± 1	33	N/A
2	NM_006842	*SF3B2*	splicing factor 3b, subunit 2, 145kDa	4 ± 1	23	1
3	NM_002005	*FES*	feline sarcoma oncogene	4 ± 1	51	19
4	NM_015000	*STK38L*	serine/threonine kinase 38 like	5 ± 0	66	N/A
5	NM_173655	*EPHA6*	EPH receptor A6	5 ± 0	46	18
6	NM_001015879	*AURKC*	aurora kinase C	5 ± 1	51	9
7	NM_198437	*AURKA*	aurora kinase A	5 ± 1	53	9
8	NM_138554	*TLR4*	toll-like receptor 4	5 ± 2	27	N/A
9	NM_001932	*MPP3*	membrane protein, palmitoylated 3 (MAGUK p55 subfamily member 3)	5 ± 1	47	12
10	NM_000141	*FGFR2*	fibroblast growth factor receptor 2	5 ± 1	51	7
11	NM_002596	*CDK18*	cyclin-dependent kinase 18	5 ± 0	55	14
12	NM_007199	*IRAK3*	interleukin-1 receptor-associated kinase 3	6 ± 2	58	20
13	NM_001024644	*XCR1*	chemokine (C motif) receptor 1	6 ± 1	48	N/A
14	NM_130901	*OTUD7A*	OTU deubiquitinase 7A	6 ± 2	23	N/A
15	NM_017431	*PRKAG3*	protein kinase, AMP-activated, gamma 3 non-catalytic subunit	7 ± 1	27	16
16	NM_007125	*UTY*	ubiquitously transcribed tetratricopeptide repeat containing, Y-linked	7 ± 2	22	N/A
17	NM_006445	*PRPF8*	pre-mRNA processing factor 8	7 ± 2	32	1
18	NM_000291	*PGK1*	phosphoglycerate kinase 1	7 ± 1	64	24
19	NM_014002	*IKBKE*	inhibitor of kappa light polypeptide gene enhancer in B-cells, kinase epsilon	7 ± 1	52	N/A
20	NM_014586	*HUNK*	hormonally up-regulated Neu-associated kinase	8 ± 0	52	N/A

Supplementary Table S7: Primary and confirmatory screening data of selected HIF-1α–NanoLuc inhibitor hits

Compound Name	HIF-1α–NanoLuc primary IC_{50}, µM (Efficacy, %)	HIF-1α–NanoLuc confirmatory IC_{50}, µM (Efficacy, %)	Viability (Luc) confirmatory IC_{50}, µM (Efficacy, %)	HRE–bla confirmatory IC_{50}, µM (Efficacy, %)	Viability (BLA) confirmatory IC_{50}, µM (Efficacy, %)	Description
Actinomycin D	0.04 (99)	0.03 ± 0.01 (87 ± 4)	Inactive	0.02 ± 0.00 (78 ± 5)	1.52 ± 2.33 (50 ± 22)	Transcription inhibitor.
AGK2	N/A	19.50 ± 2.49 (107 ± 9)	21.20 ± 3.96 (82 ± 3.96)	9.47 ± 3.45 (63 ± 14)	10.08 ± 2.97 (61 ± 13)	SIRT inhibitor.
Alexidine	10.59 (126)	9.35 ± 0.00 (107 ± 2)	18.89 ± 3.53 (115 ± 3)	8.78 ± 1.12 (87 ± 18)	8.83 ± 1.59 (102 ± 5)	Anti-microbial.
AMI-193 (Spiramide)	3.35 (57)	12.25 ± 0.83 (48 ± 6)	Inactive	Inactive	25.84 ± 4.40 (32 ± 12)	5-HT_{2A}, 5-HT_{1A}, and D_2 receptor antagonist.
5-Azacitidine	2.11 (122)	1.49 ± 0.17 (98 ± 4)	Inactive	11.01 ± 0.75 (94 ± 2)	Inactive	Cytidine analog. Drug to treat myelodysplatic syndrome.
Azathioprine	3.42 (67)	8.07 ± 1.82 (50 ± 11)	2.11 ± 0.53 (51 ± 4)	Inactive	Inactive	Anti-metabolite. Immunosuppressant.
Bisacodyl	0.84 (110)	3.91 ± 0.70 (26 ± 6)	Inactive	0.84 ± 0.10 (93 ± 4)	0.69 ± 0.05 (82 ± 3)	Laxative drug.
Camptothecin	N/A	0.57 ± 0.04 (102 ± 5)	Inactive	0.20 ± 0.06 (84 ± 1)	Inactive	Topoisomerase I inhibitor.
Canertinib	2.37 (50)	28.84 ± 10.72 (88 ± 20)	Inactive	4.67 ± 7.50 (37 ± 22)	21.21 ± 2.44 (105 ± 6)	EGFR inhibitor.
Cloflucarban	7.50 (66)	14.28 ± 0.93 (98 ± 4)	Inactive	6.80 ± 1.55 (98 ± 1)	22.94 ± 3.16 (106 ± 9)	Antiseptic.
Cycloheximide	0.24 (92)	0.53 ± 0.00 (104 ± 5)	Inactive	1.06 ± 0.12 (92 ± 2)	8.34 ± 4.61 (44 ± 15)	Protein synthesis inhibitor.
Cylcosporine A	11.88 (87)	17.38 ± 2.22 (91 ± 5)	Inactive	10.63 ± 1.22 (51 ± 3)	Inactive	Immunosuppressant.
Dabrafenib	N/A	0.47 ± 0.24 (26 ± 13)	Inactive	6.40 ± 1.55 (43 ± 1)	Inactive	B-Raf inhibitor.
Dasatinib	21.13 (56)	23.58 ± 8.59 (46 ± 3)	Inactive	0.03 ± 0.02 (52 ± 11)	Inactive	SRC/ABL inhibitor.
Daunorubicin	0.75 (51)	1.96 ± 0.35 (98 ± 8)	Inactive	1.00 ± 0.44 (80 ± 5)	3.28 ± 1.23 (75 ± 13)	DNA intercalator and topoisomerase I inhibitor.
Deslanoside	0.30 (115)	0.39 ± 0.03 (108 ± 9)	Inactive	0.16 ± 0.03 (102 ± 3)	0.32 ± 0.09 (86 ± 9)	Na^+/K^+ pump blocker.
Digitoxin	0.03 (124)	0.11 ± 0.01 (101 ± 1)	Inactive	0.03 ± .01 (97 ± 0)	0.09 ± 0.03 (91 ± 4)	Na^+/K^+ pump blocker.
Diphenoxylate	16.79 (82)	13.74 ± 0.93 (104 ± 12)	Inactive	7.80 ± 0.53 (60 ± 4)	Inactive	Centrally-active opioid drug.
Emetine	0.19 (136)	0.30 ± 0.08 (106 ± 1)	Inactive	0.29 ± 0.05 (91 ± 1)	Inactive	Anti-protozoal and a drug to induce vomiting.

Erlotinib	2.66 (33)	6.64 ± 2.42 (35 ± 6)	Inactive	7.95 ± 3.47 (59 ± 15)	Inactive	EGFR inhibitor.
Ethaverine	6.68 (67)	9.39 ± 1.08 (96 ± 2)	Inactive	Inactive	Inactive	Calcium channel blocker. Drug to treat peripheral vascular disease.
Fanetizole	26.60 (123)	16.36 ± 3.97 (28 ± 5)	Inactive	Inactive	Inactive	Immunomodulatory agent.
Gefitnib	0.19 (86)	19.41 ± 1.31 (31 ± 3)	Inactive	11.38 ± 9.73 (45 ± 14)	Inactive	EGFR inhibitor.
Lapatinib	33.49 (56)	20.17 ± 1.31 (50 ± 6)	Inactive	11.44 ± 0.75 (78 ± 6)	20.35 ± 1.33 (95 ± 7)	EGFR inhibitor.
Leflunomide	4.22 (60)	11.81 ± 6.00 (42 ± 10)	Inactive	Inactive	Inactive	Pyrimidine synthesis inhibitor.
LY-294002	N/A	14.58 ± 3.54 (50 ± 9)	Inactive	12.03 ± 2.25 (35 ± 8)	Inactive	PI3K inhibitor.
MitMAB	2.11 (129)	15.25 ± 7.16 (99 ± 9)	Inactive	5.73 ± 1.88 (100 ± 5)	18.22 ± 2.51 (57 ± 3)	Dynamin inhibitor.
Mitoxantrone	2.38 (80)	7.65 ± 2.13 (110 ± 5)	Inactive	0.86 ± 0.52 (99 ± 10)	13.38 ± 4.40 (119 ± 11)	Topoisomerase I inhibitor.
Mycophenolate mofetil	0.33 (61)	1.58 ± 0.45 (59 ± 5)	Inactive	0.21 ± 0.05 (58 ± 4)	0.53 ± 0.00 (26 ± 5)	Inosine monophosphate dehydrogenase inhibitor.
Naftopidil	7.50 (65)	9.71 ± 6.34 (43 ± 11)	Inactive	Inactive	Inactive	Alpha blocker. Anti-hypertensive drug.
Niclosamide	0.37 (115)	1.59 ± 0.40 (96 ± 5)	2.95 ± 01.93 (38 ± 4)	1.67 ± 0.46 (84 ± 6)	1.63 ± 2.58 (37 ± 9)	Anthelmintic.
Nitazoxanide	9.44 (150)	17.30 ± 1.17 (101 ± 14)	9.39 ± 1.08 (44 ± 3)	3.00 ± 0.34 (88 ± 1)	3.00 ± 0.34 (88 ± 1)	Anti-protozoal.
Ouabain	0.08 (130)	0.14 ± 0.01 (100 ± 2)	Inactive	0.08 ± 0.01 (94 ± 4)	0.13 ± 0.00 (81 ± 3)	Na^+/K^+ pump blocker.
Oxyphenisatin acetate	1.33 (84)	2.47 ± 0.79 (55 ± 9)	Inactive	0.19 ± 0.01 (92 ± 3)	0.20 ± 0.01 (77 ± 5)	Laxative.
PD-184352	0.84 (64)	13.74 ± 0.93 (99 ± 2)	Inactive	5.98 ± 0.69 (74 ± 5)	5.93 ± 4.08 (32 ± 9)	MEK inhibitor.
PI-103	N/A	0.92 ± 0.06 (57 ± 10)	Inactive	0.15 ± 0.02 (62 ± 3)	0.77 ± 0.25 (32 ± 5)	mTOR inhibitor.
PP-242	0.75 (115)	2.05 ± 1.12 (90 ± 11)	Inactive	0.05 ± 0.02 (56 ± 6)	15.05 ± 2.44 (36 ± 3)	mTOR inhibitor.
Proscillaridin A	0.01 (101)	0.01 ± 0.00 (103 ± 3)	Inactive	0.01 ± 0.00 (93 ± 3)	0.02 ± 0.00 (86 ± 6)	Na^+/K^+ pump blocker.
Quercetine	0.84 (44)	37.22 ± 0.00 (37 ± 1)	Inactive	12.84 ± 0.84 (84 ± 10)	Inactive	HDAC inhibitor.
Selumetinib	0.04 (62)	1.49 ± 1.30 (48 ± 10)	Inactive	Inactive	Inactive	MEK inhibitor.
Sorafenib	13.33 (156)	23.59 ± 2.71 (100 ± 6)	Inactive	17.59 ± 8.60 (98 ± 7)	Inactive	VEGFR/PDGFR/Raf inhibitor.
SU-5416	23.71 (67)	12.23 ± 7.55 (57 ± 22)	Inactive	12.84 ± 0.84 (111 ± 12)	Inactive	VEGFR inhibitor.

Sunitinib	1.68 (60)	11.34 ± 3.71 (80 ± 15)	26.36 ± 9.41 (26 ± 7)	16.99 ± 3.17 (81 ± 32)	15.98 ± 8.13 (60 ± 38)	VEGFR inhibitor.
Temsirolimus	N/A	34.52 ± 2.34 (92 ± 8)	Inactive	15.86 ± 6.91 (86 ± 7)	27.66 ± 1.87 (116 ± 11)	mTOR inhibitor.
6-Thioguanine (6-TG)	2.11 (81)	3.26 ± 0.81 (81 ± 7)	2.30 ± 0.87 (57 ± 2)	7.83 ± 1.00 (88 ± 1)	Inactive	Anti-metabolite. Drug to treat leukemia.
Topotecan	6.68 (98)	2.10 ± 0.24 (99 ± 6)	Inactive	0.62 ± 0.38 (131 ± 9)	Inactive	Topoisomerase I inhibitor.
Trametinib	N/A	2.98 ± 4.64 (69 ± 5)	Inactive	20.51 ± 8.11 (53 ± 9)	Inactive	MEK inhibitor.
Trichostatin A	N/A	0.67 ± 0.26 (55 ± 4)	Inactive	12.19 ± 3.90 (92 ± 11)	9.68 ± 3.79 (43 ± 13)	HDAC inhibitor.
Vemurafenib	N/A	13.59 ± 6.38 (66 ± 6)	Inactive	4.10 ± 0.70 (75 ± 11)	15.98 ± 8.13 (27 ± 10)	B-Raf inhibitor.
Wortmannin	N/A	0.99 ± 0.22 (71 ± 4)	Inactive	0.57 ± 0.17 (91 ± 8)	11.41 ± 4.79 (47 ± 11)	PI3K inhibitor.

Supplementary Table S8: Cancer cell proliferation data of selected HIF-1α–NanoLuc inhibitors

Compound Name	HCT116 3-Day Viability IC_{50}, µM (Efficacy, %)	ME-180 3-Day Viability IC_{50}, µM (Efficacy, %)	HIF-1α–NanoLuc Activity/HCT116 3-Day Viability	HIF-1α–NanoLuc Activity/ME-180 3-Day Viability
5-Azacitidine	13.27 ± 1.52 (90 ± 9)	18.65 ± 0.00 (51 ± 12)	0.1	0.08
Camptothecin	1.00 ± 0.29 (59 ± 9)	0.04 ± 0.00 (100 ± 2)	1.5	14
Daunorubicin	2.43 ± 1.40 (79 ± 28)	0.17 ± 0.08 (101 ± 4)	0.8	0.8
Deslanoside	2.59 ± 2.87 (63 ± 8)	0.31 ± 0.04 (97 ± 3)	0.2	12
Digitoxin	2.29 ± 3.13 (97 ± 8)	0.09 ± 0.02 (101 ± 4)	0.05	1.2
LY-294002	Inactive	15.42 ± 1.04 (73 ± 4)	N/A	0.9
Mitoxantrone	11.10 ± 4.79 (80 ± 5)	0.11 ± 0.03 (97 ± 4)	0.7	69
Mycophenolate mofetil	Inactive	1.82 ± 0.74 (81 ± 3)	N/A	0.9
Niclosamide	1.67 ± 0.46 (83 ± 6)	2.04 ± 0.00 (86 ± 1)	1.0	0.8
Ouabain	0.63 ± 0.37 (73 ± 13)	0.13 ± 0.01 (98 ± 4)	0.2	1.1
PD-184352	13.21 ± 0.00 (97 ± 4)	17.98 ± 1.17 (100 ± 7)	1.0	0.8
PI-103	Inactive	0.37 ± 0.02 (85 ± 5)	N/A	2.5
PP-242	33.60 ± 6.28 (49 ± 20)	0.95 ± 0.22 (105 ± 7)	0.1	2.2
Proscillaridin A	0.09 ± 0.03 (87 ± 20)	0.02 ± 0.00 (98 ± 2)	0.1	0.5
Selumetinib	22.83 ± 3.89 (105 ± 4)	0.27 ± 0.06 (36 ± 3)	0.07	5.5
Sunitinib	Inactive	18.99 ± 4.34 (90 ± 7)	N/A	0.6
Topotecan	4.30 ± 1.20 (54 ± 22)	0.10 ± 0.01 (100 ± 2)	0.5	21
Trametinib	0.64 ± 0.11 (87 ± 3)	0.06 ± 0.02 (52 ± 7)	5	50
Vemurafenib	31.79 ± 9.41 (63 ± 4)	14.35 ± 1.97 (92 ± 5)	0.4	0.9
Wortmannin	Inactive	7.00 ± 4.33 (80 ± 3)	N/A	0.1

Supplementary Table S9: List of human hypoxia signaling genes including HIF-1 and its co-transcription factors, other HIF-1 interacting proteins, and responsive genes (i.e., angiogenesis, coagulation, DNA damage and repair, metabolism, apoptosis, cell proliferation, transcription factors, transporters/channels/receptors, and other)

Symbol	Refseq	Gene Description	Function Class
ADM	NM_001124	Adrenomedullin	Apoptosis & cell proliferation
ADORA2B	NM_000676	Adenosine A2b receptor	Angiogenesis
ALDOA	NM_000034	Aldolase A, fructose-bisphosphate	Coagulation & metabolism
ANGPTL4	NM_001039667	Angiopoietin-like 4	Angiogenesis
ANKRD37	NM_181726	Ankyrin repeat domain 37	Other responsive genes
ANXA2	NM_004039	Annexin A2	Angiogenesis & coagulation
APEX1	NM_080649	APEX nuclease (multifunctional DNA repair enzyme) 1	Other HIF-1 interactors
ARNT	NM_001668	Aryl hydrocarbon receptor nuclear translocator	HIF-1 & co-transcription factors
ATR	NM_001184	Ataxia telangiectasia and Rad3 related	DNA damage and repair
BHLHE40	NM_003670	Basic helix-loop-helix family, member e40	Transcription factors
BLM	NM_000057	Bloom syndrome, RecQ helicase-like	Cell proliferation
BNIP3	NM_004052	BCL2/adenovirus E1B 19kDa interacting protein 3	Apoptosis
BNIP3L	NM_004331	BCL2/adenovirus E1B 19kDa interacting protein 3-like	Apoptosis
BTG1	NM_001731	B-cell translocation gene 1, anti-proliferative	Angiogenesis, apoptosis & cell proliferation
CA9	NM_001216	Carbonic anhydrase IX	Other responsive genes
CCNG2	NM_004354	Cyclin G2	Cell proliferation
COPS5	NM_006837	COP9 constitutive photomorphogenic homolog subunit 5 (Arabidopsis)	HIF-1 & co-transcription factors
CTSA	NM_000308	Cathepsin A	Other responsive genes
DDIT4	NM_019058	DNA-damage-inducible transcript 4	Metabolism & apoptosis
DNAJC5	NM_025219	DnaJ (Hsp40) homolog, subfamily C, member 5	Other responsive genes
EDN1	NM_001955	Endothelin 1	Angiogenesis
EGLN1	NM_022051	Egl nine homolog 1 (C. elegans)	Other HIF-1 interactors
EGLN2	NM_053046	Egl nine homolog 2 (C. elegans)	Other HIF-1 interactors
EGR1	NM_001964	Early growth response 1	Angiogenesis & cell proliferation
EIF4EBP1	NM_004095	Eukaryotic translation initiation factor 4E binding protein 1	Other responsive genes
ENO1	NM_001428	Enolase 1, (alpha)	Metabolism
EPO	NM_000799	Erythropoietin	Angiogenesis
ERO1L	NM_014584	ERO1-like (S. cerevisiae)	Metabolism
F10	NM_000504	Coagulation factor X	Coagulation
F3	NM_001993	Coagulation factor III (thromboplastin, tissue factor)	Angiogenesis & coagulation
FOS	NM_005252	FBJ murine osteosarcoma viral oncogene homolog	Transcription factors
GBE1	NM_000158	Glucan (1, 4-alpha-), branching enzyme 1	Metabolism
GPI	NM_000175	Glucose-6-phosphate isomerase	Angiogenesis & metabolism
GYS1	NM_002103	Glycogen synthase 1 (muscle)	Metabolism

HIF1A	NM_001530	Hypoxia inducible factor 1, alpha subunit (basic helix-loop-helix transcription factor)	HIF-1 & co-transcription factors
HIF1AN	NM_017902	Hypoxia inducible factor 1, alpha subunit inhibitor	HIF-1 & co-transcription factors
HIF3A	NM_152794	Hypoxia inducible factor 3, alpha subunit	HIF-1 & co-transcription factors
HK2	NM_000189	Hexokinase 2	Metabolism
HMOX1	NM_002133	Heme oxygenase (decycling) 1	Angiogenesis
HNF4A	NM_178849	Hepatocyte nuclear factor 4, alpha	HIF-1 & co-transcription factors
IER3	NM_003897	Immediate early response 3	Apoptosis
IGFBP3	NM_000598	Insulin-like growth factor binding protein 3	Cell proliferation
JMJD6	NM_015167	Jumonji domain containing 6	Angiogenesis
LDHA	NM_005566	Lactate dehydrogenase A	Metabolism
LGALS3	NM_002306	Lectin, galactoside-binding, soluble, 3	Other responsive genes
LOX	NM_002317	Lysyl oxidase	Angiogenesis
MAP3K1	NM_005921	Mitogen-activated protein kinase kinase kinase 1	Other responsive genes
MET	NM_000245	Met proto-oncogene (hepatocyte growth factor receptor)	Cell proliferation
MIF	NM_002415	Macrophage migration inhibitory factor (glycosylation-inhibiting factor)	DNA damage and repair & apoptosis & cell proliferation
MMP9	NM_004994	Matrix metallopeptidase 9 (gelatinase B, 92kDa gelatinase, 92kDa type IV collagenase)	Angiogenesis
MXI1	NM_005962	MAX interactor 1	Cell proliferation
NAMPT	NM_005746	Nicotinamide phosphoribosyltransferase	Cell proliferation
NCOA1	NM_003743	Nuclear receptor coactivator 1	HIF-1 & co-transcription factors
NDRG1	NM_006096	N-myc downstream regulated 1	DNA damage and repair
NFKB1	NM_003998	Nuclear factor of kappa light polypeptide gene enhancer in B-cells 1	Other HIF-1 interactors
NOS3	NM_000603	Nitric oxide synthase 3 (endothelial cell)	Apoptosis & cell proliferation
ODC1	NM_002539	Ornithine decarboxylase 1	Cell proliferation
P4HA1	NM_000917	Prolyl 4-hydroxylase, alpha polypeptide I	Other HIF-1 interactors
P4HB	NM_000918	Prolyl 4-hydroxylase, beta polypeptide	Other HIF-1 interactors
PDK1	NM_002610	Pyruvate dehydrogenase kinase, isozyme 1	Metabolism
PER1	NM_002616	Period homolog 1 (Drosophila)	HIF-1 & co-transcription factors
PFKFB3	NM_004566	6-phosphofructo-2-kinase/fructose-2, 6-biphosphatase 3	Metabolism
PFKFB4	NM_004567	6-phosphofructo-2-kinase/fructose-2, 6-biphosphatase 4	Metabolism
PFKL	NM_002626	Phosphofructokinase, liver	Metabolism
PFKP	NM_002627	Phosphofructokinase, platelet	Metabolism
PGAM1	NM_002629	Phosphoglycerate mutase 1 (brain)	Metabolism
PGF	NM_002632	Placental growth factor	Angiogenesis & cell proliferation
PGK1	NM_000291	Phosphoglycerate kinase 1	Metabolism
PIM1	NM_002648	Pim-1 oncogene	Apoptosis & cell proliferation
PKM	NM_002654	Pyruvate kinase, muscle	Metabolism
PLAU	NM_002658	Plasminogen activator, urokinase	Angiogenesis & coagulation
RBPJ	NM_005349	Recombination signal binding protein for immunoglobulin kappa J region	Transcription factors

RUVBL2	NM_006666	RuvB-like 2 (E. coli)	DNA damage and repair
SERPINE1	NM_000602	Serpin peptidase inhibitor, clade E (nexin, plasminogen activator inhibitor type 1), member 1	Angiogenesis & coagulation
SLC16A3	NM_004207	Solute carrier family 16, member 3 (monocarboxylic acid transporter 4)	Coagulation
SLC2A1	NM_006516	Solute carrier family 2 (facilitated glucose transporter), member 1	Metabolism & transporters, channels, and receptors
SLC2A3	NM_006931	Solute carrier family 2 (facilitated glucose transporter), member 3	Metabolism & transporters, channels, and receptors
TFRC	NM_003234	Transferrin receptor (p90, CD71)	Transporters, channels, and receptors
TP53	NM_000546	Tumor protein p53	Other HIF-1 interactors
TPI1	NM_000365	Triosephosphate isomerase 1	Metabolism
TXNIP	NM_006472	Thioredoxin interacting protein	Cell proliferation
USF2	NM_003367	Upstream transcription factor 2, c-fos interacting	Transcription factors
VDAC1	NM_003374	Voltage-dependent anion channel 1	Transcription factors & transporters, channels, and receptors
VEGFA	NM_003376	Vascular endothelial growth factor A	Angiogenesis

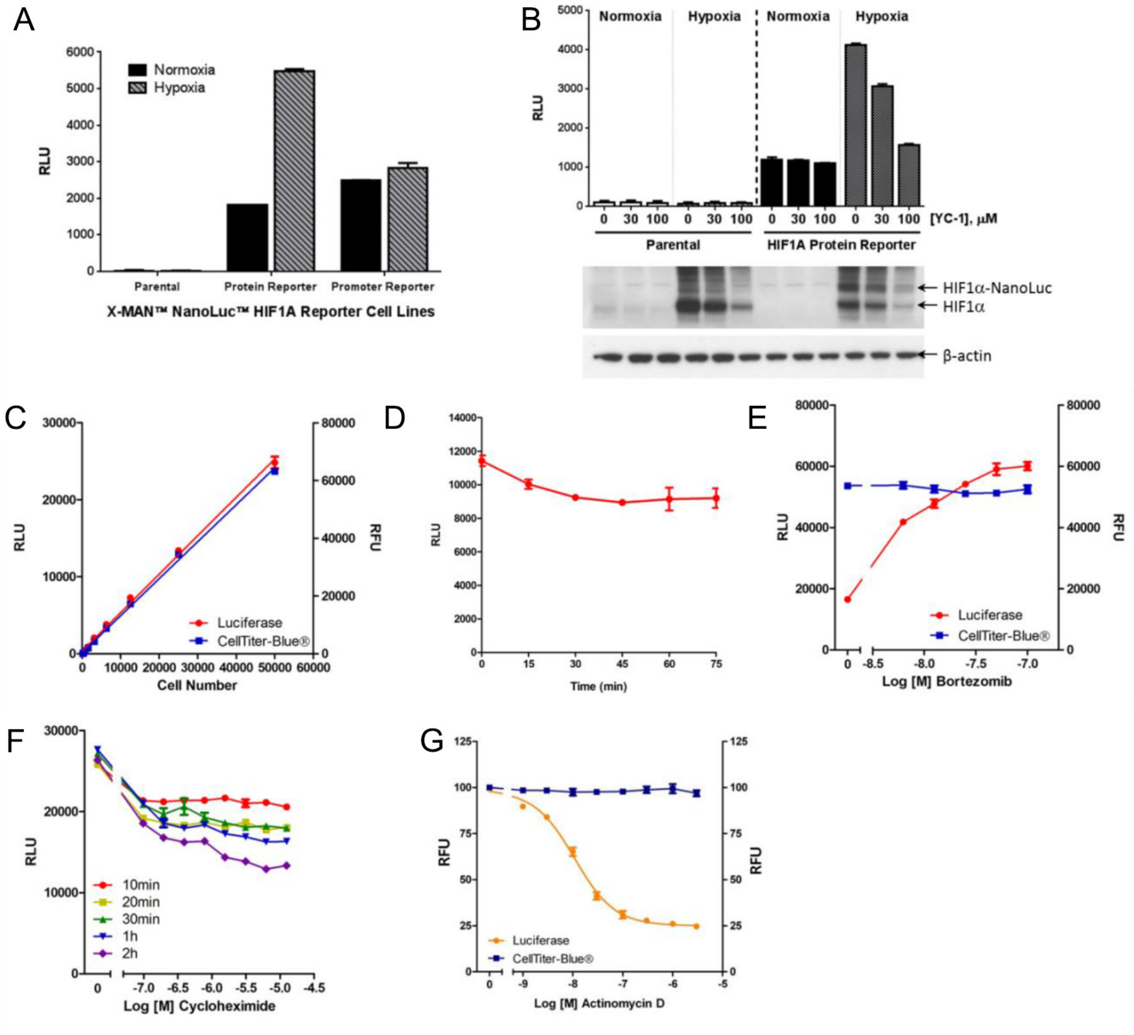

Supplementary Figure S1: Development and validation of HIF-1α–NanoLuc assay. (**A**) A three-fold increase in hypoxia-induced luciferase activity of HIF-1α-NanoLuc protein reporter cells was observed after exposing cells to 1% O_2 (hypoxia) and 20% O_2 (normoxia) for four hours. (**B**) *Top*: Under hypoxic conditions HIF-1α-NanoLuc HCT116 cells responded to the HIF-1 inhibitor (YC-1) with a decrease in luciferase expression in a concentration response manner. *Bottom*: The increased levels of HIF-1α protein under the same hypoxic treatment were evident on Western blots in both parental and HIF-1α-NanoLuc cells. (**C**) The assay exhibited a linear correlation between luciferase signals and cell number. (**D**) The assay has stable decay kinetics. (**E**) Exposure of HIF-1α-NanoLuc cells to a proteasome inhibitor (bortezomib) resulted in protein accumulation and increased luciferase activity. (**F**) HIF-1α-NanoLuc cells treated with cycloheximide showed reduced protein turnover and decreased luciferase signal. (**G**) Treatment of HIF-1α-NanoLuc cells with a transcription inhibitor (actinomycin) caused reduced NanoLuc activity.

For western blot analysis, HCT116 and HIF-1α–NanoLuc cells were seeded into 6-well plates and incubated with YC-1 at 37ºC, 5% CO_2 under normoxic or hypoxic (1% O_2) conditions for four hours. Cells were lysed in Tris-Glycine buffer and the protein concentration was determined using the BCA protein assay (Thermo Fisher Scientific). Proteins loaded onto 10% Bis-Tris gels were resolved by SDS-PAGE and transferred onto PVDF membranes (Life Technologies). Anti-HIF-1α antibody (1:500; BD Transduction Labs), anti-β-actin antibody (1:500,000; Sigma-Aldrich), appropriate secondary antibodies (Cell Signaling Technology), and ECL-Plus detection reagent (GE Healthcare Bio-Sciences) were used to immunoblot protein levels of the corresponding targets according manufacturer's protocols.

A

Curve Class	Efficacy	Curve	Curve fit
-1.1 & 1.1	High	Complete	Good
-1.2 & 1.2	Partial	Complete	Good
-1.3 & 1.3	High	Complete	Poor
-1.4 & 1.4	Partial	Complete	Poor
-2.1 & 2.1	High	Partial	Good
-2.2 & 2.2	Partial	Partial	Good
-2.3 & 2.3	High	Partial	Poor
-2.4 & 2.4	Partial	Partial	Poor
-3 & 3	Single point of Activity		
4	Inactive		

B

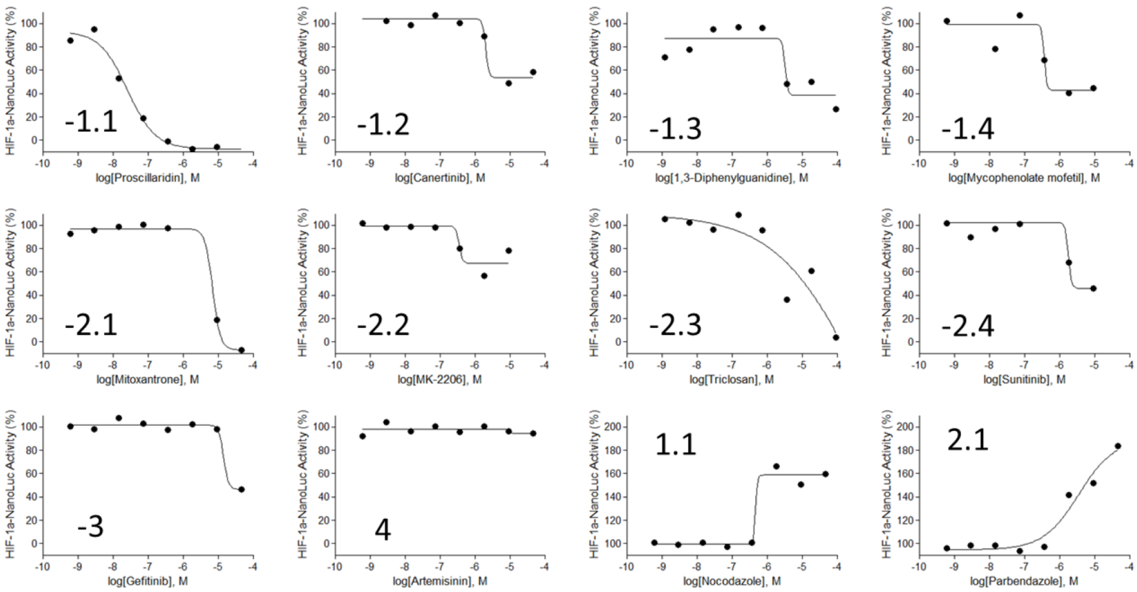

Supplementary Figure S2: Concentration-response curves and curve classes of selected HIF-1α–NanoLuc inhibitor hits identified from primary screening. (**A**) Categories of curve classes based on efficacy, curve completeness, and goodness of curve fit. Inactive compounds were assigned to curve class 4. Other compounds with positive curve classes are activators and ones with negative curve classes are inhibitors. (**B**) Examples of identified HIF-1α–NanoLuc hits representation each curve class.

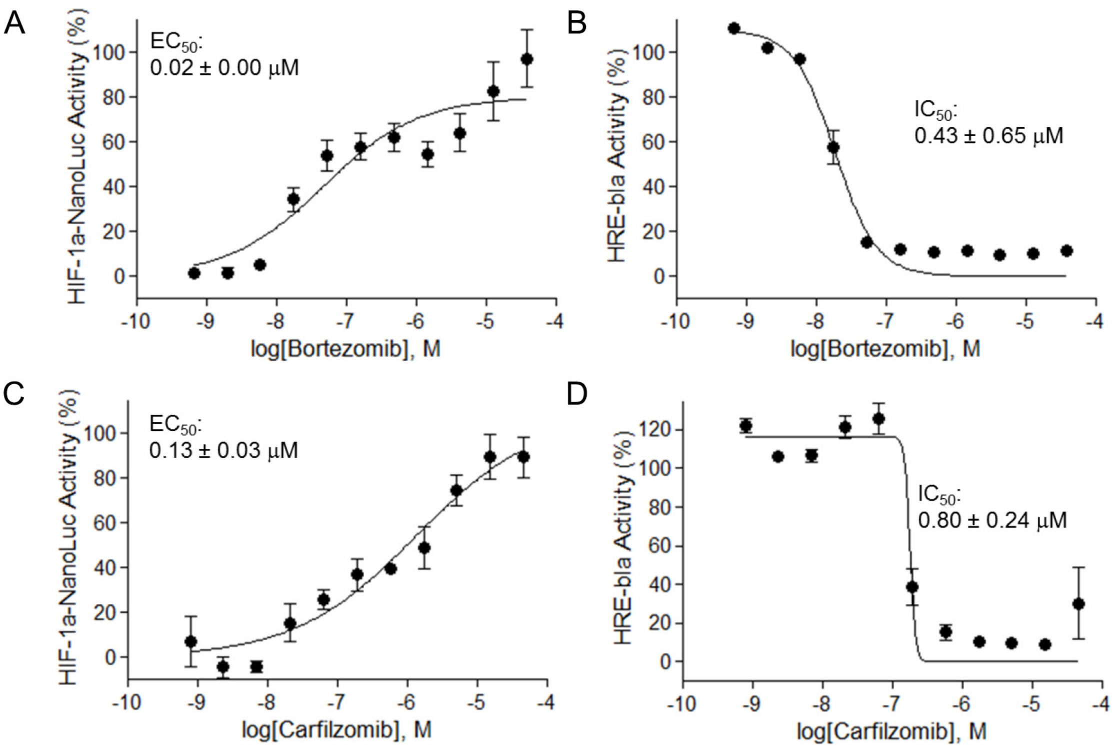

Supplementary Figure S3: Concentration-response curves of bortezomib and carfilzomib measured in the HIF-1α–NanoLuc and the HRE–bla confirmatory screens. (**A**) Bortezomib in HIF-1α–NanoLuc assay. (**B**) Bortezomib in HRE–bla assay. (**C**) Carfilzomib in HIF-1α–NanoLuc assay. (**D**) Carfilzomib in HRE–bla assay. The data was obtained from three independent measurements and represented as mean ± standard deviation.

REFERENCES

1. Nagle DG, Zhou YD. Natural product-based inhibitors of hypoxia-inducible factor-1 (HIF-1). Curr Drug Targets. 2006; 7:355–369.
2. Semenza GL. Evaluation of HIF-1 inhibitors as anticancer agents. Drug Discov Today. 2007; 12:853–859.
3. Oda S, Oda T, Takabuchi S, Nishi K, Wakamatsu T, Tanaka T, Adachi T, Fukuda K, Nohara R, Hirota K. The calcium channel blocker cilnidipine selectively suppresses hypoxia-inducible factor 1 activity in vascular cells. Eur J Pharmacol. 2009; 606:130–136.
4. Lee K, Qian DZ, Rey S, Wei H, Liu JO, Semenza GL. Anthracycline chemotherapy inhibits HIF-1 transcriptional activity and tumor-induced mobilization of circulating angiogenic cells. Proc Natl Acad Sci U S A. 2009; 106:2353–2358.
5. Zhang H, Qian DZ, Tan YS, Lee K, Gao P, Ren YR, Rey S, Hammers H, Chang D, Pili R, Dang CV, Liu JO, Semenza GL. Digoxin and other cardiac glycosides inhibit HIF-1alpha synthesis and block tumor growth. Proc Natl Acad Sci U S A. 2008; 105:19579–19586.
6. Park HJ, Kim MS, Cho K, Yun JH, Choi YJ, Cho CH. Disulfiram deregulates HIF-alpha subunits and blunts tumor adaptation to hypoxia in hepatoma cells. Acta Pharmacol Sin. 2013; 34:1208–1216.
7. Zhou YD, Kim YP, Mohammed KA, Jones DK, Muhammad I, Dunbar DC, Nagle DG. Terpenoid tetrahydroisoquinoline alkaloids emetine, klugine, and isocephaeline inhibit the activation of hypoxia-inducible factor-1 in breast tumor cells. J Nat Prod. 2005; 68:947–950.
8. Newcomb EW, Ali MA, Schnee T, Lan L, Lukyanov Y, Fowkes M, Miller DC, Zagzag D. Flavopiridol downregulates hypoxia-mediated hypoxia-inducible factor-1alpha expression in human glioma cells by a proteasome-independent pathway: implications for in vivo therapy. Neuro Oncol. 2005; 7:225–235.
9. Toh YM, Li TK. Mitoxantrone inhibits HIF-1alpha expression in a topoisomerase II-independent pathway. Clin Cancer Res. 2011; 17:5026–5037.
10. Pore N, Gupta AK, Cerniglia GJ, Maity A. HIV protease inhibitors decrease VEGF/HIF-1alpha expression and angiogenesis in glioblastoma cells. Neoplasia. 2006; 8:889–895.
11. Liu LP, Ho RL, Chen GG, Lai PB. Sorafenib inhibits hypoxia-inducible factor-1alpha synthesis: implications for antiangiogenic activity in hepatocellular carcinoma. Clin Cancer Res. 2012; 18:5662–5671.
